# Effects of the Lipid Metabolites and the Gut Microbiota in ApoE^−/−^ Mice on Atherosclerosis Co-Depression From the Microbiota-Gut-Brain Axis

**DOI:** 10.3389/fmolb.2022.786492

**Published:** 2022-04-26

**Authors:** Ke Hu, Xing-Xing Liao, Xiao-Yun Wu, Rui Wang, Zi-Wei Hu, Si-Yuan Liu, Wen-Fen He, Jun-Jie Zhou

**Affiliations:** ^1^ School of Rehabilitation Medicine, Gannan Medical University, Ganzhou, China; ^2^ School of Basic Medicine, Gannan Medical University, Ganzhou, China; ^3^ Key Laboratory of Prevention and Treatment of Cardiovascular and Cerebrovascular Diseases of Ministry of Education, Gannan Medical University, Ganzhou, China

**Keywords:** atherosclerosis, depression, microbiota-gut-brain axis, gut microbiota, lipid metabolites, ApoE^−/−^

## Abstract

**Background:** The diagnosis, treatment, and prevention of atherosclerosis co-depression are poor, so it is urgent to explore new targets. Based on the “microbiota-gut-brain axis,” this study aimed to investigate the changes of lipid metabolites in the prefrontal cortex and hippocampus regions and the characteristics of the gut microbiota in ApoE^−/−^ mice with atherosclerosis co-depression.

**Methods:** ApoE^−/−^ mice (hyperlipid feeding combined with binding, HFB group, *n* = 14, male) fed a high-fat diet for 16 weeks with binding stimulation were used as an animal model for atherosclerosis co-depression. The depression degree of mice was evaluated by body weight, sucrose preference test, open field test, and tail suspension test. Oil-red O staining, HE staining, and biochemical parameters were used to evaluate the damage degree of atherosclerosis in mice. LC-MS/MS technique for non-targeted lipidomics analysis was used to analyze the differential lipid metabolites in the prefrontal cortex and hippocampus regions of mice. 16S rDNA amplification sequencing was used to screen the differential gut microbial, and association analysis was performed with the differential lipid metabolites.

**Results:** Compared with the normal control group (NC group), the HFB group showed depression-like behaviors and atherosclerosis-related pathological indicators. The differential lipid metabolites in the prefrontal cortex and hippocampus regions were mainly LPC, LPE, LPS, PC, PE, PS, PI, and GD1a, and were mainly enriched in the glycerophospholipid metabolism pathway and the retrograde endocannabinoid signaling pathway. At the same time, there were significant differences in the structure of the gut microbial community between the two groups. The abundance of *Deferribacteres* and *Proteobacteria* in the HFB group increased, while the abundance of *Verrucomicrobia* and *Actinobacteria* decreased at the phylum level; the abundance of *Desulfovibrio*, *Clostridium_IV*, *Helicobacter* and *Pseudoflavonifractor* increased, while the abundance of *Akkermansia* decreased at the genus level.

**Conclusion:** Atherosclerosis co-depression of ApoE^−/−^ mice of the prefrontal cortex and hippocampus lipid metabolism pathways of disorder and the changes of to the gut microbiota, which leads to abnormal white matter and synaptic dysfunction, increased gut inflammation, and decreased gut permeability, leading to the release of inflammatory cytokines, there is a strong correlation between both, it further confirmed the existence of the “microbiota-gut-brain axis.”

## 1 Introduction

Cardiovascular and cerebrovascular diseases caused by atherosclerosis, such as coronary heart disease, cerebral infarction, peripheral vascular disease, have become one of the important causes of serious harm to human life and health (Zhu et al., 2018; Libby et al., 2019). Depression is a kind of mood disorder whose main clinical feature is significant and persistent low mood (Hao et al., 2019). In the relevant report of the American Heart Association in 2014, depression was listed as an independent risk factor for the incidence of coronary heart disease and the quality of prognosis (Lichtman et al., 2014). In clinical practice, cardiologists have a high rate of missed diagnosis for heart disease combined with psychological disease (Jha et al., 2019). They often use western drugs to treat coronary heart disease combined with antidepressant treatment, but there is a problem of long-term drug toxicity and side effects (Wu et al., 2020). At present, the pathogenesis between the two diseases has not been fully defined. In terms of basic research, there are few studies on the establishment and evaluation of the atherosclerosis co-depression mouse model, which limits the follow-up drug intervention studies on it. In our published paper, we demonstrated that ApoE^−/−^ mice with a high-fat diet accelerated the formation of atherosclerosis and depression-like behavior changes. RNA-seq combined with bioinformatics analysis showed that inflammatory chemokine genes and related pathways are highly expressed in the prefrontal cortex and hippocampus regions of the atherosclerosis co-depression mice, but the detailed mechanisms affecting these genes and pathways need to be further studied (Zhou et al., 2019).

With the proposed theory of the “microbiota-gut-brain axis” (Cryan et al., 2019), the microbiota can affect the function and behavior of brain regions through the gut metabolism process, leading to the occurrence of diseases (Romaní-Pérez et al., 2021). More and more studies have confirmed that there is a strong correlation between gut microbiota and cardiovascular diseases and depression. The imbalance of the gut microbiota function and the changes in related metabolic patterns are closely related to the pathogenesis of the disease (Lindskog Jonsson et al., 2018; Chi et al., 2020; Flux and Lowry, 2020; Wu et al., 2020). According to a new study published in Nature, the microbiota can regulate social behavior through stress-responding neurons in the brain (Wu et al., 2021). Therefore, there must be a correlation between gut microbiota and brain metabolism in mice. Studies have shown that inflammation and vascular lipid accumulation are considered to be important factors promoting the formation and development of atherosclerosis plaque (Libby et al., 2019). And the onset of depression is closely related to the functions of the prefrontal cortex and hippocampus regions (Chi et al., 2020). Then, does the occurrence of atherosclerosis co-depression affect the lipid metabolism of related brain areas through changes in the gut microbiota?

In this study, ApoE^−/−^ mice were fed a continuous high-fat diet combined with binding stimulation, and an animal model of atherosclerosis co-depression was established based on the “microbiota-gut-brain axis” theory. The LC-MS/MS technique was used for non-targeted lipidomics analysis to identify the differential lipid metabolites and major metabolic pathways in the prefrontal cortex and hippocampus regions, and the 16S recombinant-DNA (rDNA) amplification sequencing technique was used to study the changes of the gut microbiota in ApoE^−/−^ mice with atherosclerosis co-depression. Finally, we further studied the relationship between the changes of the gut microbiota and the lipid metabolisms phenotype of brain areas in ApoE^−/−^ mice with atherosclerosis co-depression by the association analysis. These findings reveal that gut microbiota is involved in the pathogenesis of atherosclerosis co-depression, expound the influence of psychological diseases on heart disease, and provide basic research for clinical intervention in atherosclerosis co-depression.

## 2 Materials and Methods

### 2.1 Experimental Animals, Grouping and Model Preparation

14 C57BL/6 mice (7 weeks old, 20 ± 2 g, male, SPF grade) and 28 ApoE^−/−^ mice (7 weeks old, 20 ± 2 g, male, SPF grade) were purchased from Beijing Weishang Lituo Technology Co., Ltd. Animal care procedures were performed under the following conditions: temperature (22°C ± 1°C), humidity (55% ± 5%), free food and water drink, alternating lighting of 12 h. The formulation of high lipid feed is 84% base feed, 10% fats, 1.5% cholesterol, 0.5% sodium cholate, 4% milk powder. The high lipid feed and ordinary feed were purchased from Nanjing CZR Technology Co., Ltd.

All animals were acclimatized for 1 week, and then they were divided into cages according to the statistical random grouping principle. 14 C57BL/6 mice for normal control (NC group, *N* = 14); 28 ApoE^−/−^ mice divides into two groups: hyperlipid feeding (HF group, *N* = 14) and hyperlipid feeding combined with binding (HFB group, *N* = 14). The mice in the HFB group were fed with a high-fat diet and trapped in a self-made 50 ml centrifuge tube per day for 1-h, continue for 16 weeks to establish atherosclerotic comorbidity with the depression model. The HF group was fed with high-fat feed but not bound, and the NC group was fed with ordinary feed but not bound.

### 2.2 Experimental Evaluation of Depressive Behavior

#### 2.2.1 Bodyweight and Sucrose Preference Test

The mice in each group were weighed every 2 weeks, and the sucrose preference test (Dans et al., 2013) was performed every 4 weeks as a sign of the progress of depression-like behaviors. The specific operation of the sucrose preference test: prepare one bottle of 1% sucrose solution and one bottle of pure water. The positions of the two bottles are changed every 12 h. The food intake during adaptation is not restricted. After 24 h of adaptation, the formal experiment will be started. Finally, weigh the amount of water and sucrose solution consumed by each group. Calculation formula: sugar preference ratio (%) = sucrose consumption/(sucrose consumption + pure water consumption) × 100%.

#### 2.2.2 Open Field Test

Open field test (Seibenhener and Wooten, 2015) is an important index to evaluate the spontaneous motor ability of mice. The mice in each group conduct an open field test every 4 weeks. Before the test, the mice were placed in the behavioral test room for 30 min to adapt to the environment. During the test, the mice were gently placed in the middle of the open field with a size of 25 cm × 25 cm × 35 cm, and the animal behavioral analysis system was used to film and record the mice after 30 s of acclimatization, and the total distance traveled and the time spent in the central area of the open field box within 250 s was measured.

#### 2.2.3 Tail Suspension Test

Tail suspension test (Can et al., 2012) evaluates the despairing behavior of mice by measuring their immobile time. The tail suspension test begins 1 week before the end of the experiment. The tails of the mice were fixed to the hooks of the test chamber with medical tape, so that the mice head is hung upside down in the test box, using the animal behavior analysis system to record the activity in 6 min of mice, and analyze the cumulative immobile time of each group in 4 min.

### 2.3 Sample Collection and Processing

#### 2.3.1 Collection of Feces

One morning before the end of the experiment period, the feces of six mice in each group were collected. Each mice collected three to five pieces of feces into a 1.5 ml enzyme-free centrifuge tube and labeled them. After being frozen in liquid nitrogen, the feces were stored in the ultra-low temperature refrigerator at −80°C for 16S rDNA extraction and analysis.

#### 2.3.2 Blood Picking

Mice were fasted for 8 h at the end of the experimental cycle and given 5% sucrose water as energy support. After inhalation anesthesia, blood samples were immediately collected from the eye socket using a 1.5 ml Eppendorf tube, placed at 4°C for 1 h, 860 × g for 15 min, centrifuged, and then the supernatant was taken and stored in an ultra-low temperature refrigerator at −80°C for subsequent biochemical parameters detection.

#### 2.3.3 Prefrontal Cortex and Hippocampus Tissue

The whole brain of mice was removed and placed on ice, prefrontal cortex and hippocampus tissues were rapidly isolated, rinsed with low-temperature saline, blotted dry on filter paper, weighed, placed in labeled cryotubes, rapidly frozen in liquid nitrogen, and stored in an ultra-low temperature refrigerator at −80°C for subsequent lipid metabolomics studies.

#### 2.3.4 Aortic

The mice were fixed on a dissecting table, perfused with 0.9% saline solution and 4% paraformaldehyde solution, the lung lobes were removed, the vessels above the plane of the diaphragm of the aortic arch were fully exposed, the surface tissue of the vessels was wiped away, the vessels were stripped from bottom to top along the spine with ophthalmic curved forceps, without damaging the endothelium as far as possible. Next, flush it at low-temperature physiological saline, use filter paper to absorb moisture. Store in labeled 2.0 ml cryopreservation tube containing 4% paraformaldehyde.

### 2.4 Atherosclerosis Model Evaluation Methods

#### 2.4.1 Oil-Red O Staining to Detect Gross Structural Changes in the Thoracic Aorta of Mice

The mice’s aortic arch was dissected lengthwise with anatomical scissors, and as much fat tissue was removed with forceps. The dissected aortic arch was immersed in Oil Red staining solution at 37° for 60 min, and then taken out, fractionated with 75% ethanol until the fatty plaques in the lumen were orange or bright red and the other parts were nearly colorless, then washed twice with distilled water, and finally, the stained bulk tissue was unfolded and fixed and photographed. Positive staining was seen for lipids showing orange or bright red in the inner wall of the aortic arch and nearly colorless elsewhere. The lesion areas were analyzed using Image-J 1.53c software.

#### 2.4.2 HE Staining to Detect Morphological Changes in Coronary Artery Histology

Direct staining of pathological sections of aortic arch tissue was performed according to the manufacturer’s instructions (G1003, Servicebio, China). Morphological changes of endothelial cells, smooth muscle cells, and foam cells on aortic arch tissue were observed under a light microscope. Changes in intima-media thickness of the aortic arch in mice were analyzed using Image-Pro Plus 6.0 software.

#### 2.4.3 Biochemical Parameters

The total cholesterol (TC), triglyceride (TG), high-density lipoprotein cholesterol (HDL-C), and low-density lipoprotein cholesterol (LDL-C) in the serum of each group of mice were measured according to the instructions of the kit (20210508, Jiancheng Bioengineering Institute, Nanjing, China).

### 2.5 Lipid Metabolomic Analysis of Mice Prefrontal Cortex and Hippocampus Tissue

#### 2.5.1 Sample Preparation

After slow thawing at 4°C, 25 mg of each sample of prefrontal cortex or hippocampus tissue was weighed into a 1.5 ml Eppendorf tube, 800 μl of extraction solution dichloromethane/methanol (3:1, v:v, pre-chilled at −20°C) and 10 μl of SPLASH internal standard reservoir were added, two small steel balls were added, and grinding in a tissue grinder (50 Hz, 5 min), sonicated in a water bath at 4°C for 10 min, and then left for 1 h in a refrigerator at −20°C. The supernatant was centrifuged at 1,520 × g for 15 min at 4°C. After centrifugation, 600 μl of the supernatant was placed in a freezing vacuum concentrator and drained. 200 μl of the solution (isopropanol: acetonitrile: H_2_O = 2:1:1, v:v:v) was added for re-dissolution, a vortex was shaken for 1 min, sonicated in a water bath at 4°C for 10 min, then centrifuged at 4°C at 1,520 × g for 15 min, and the supernatant was placed in a supernatant bottle. The supernatant of each sample was mixed into a quality control (QC) sample of 20 μl each and used to assess the reproducibility and stability of the LC-MS analysis process.

#### 2.5.2 LC-MS/MS Technique for Non-Targeted Lipidomics Analysis Conditions

In this experiment, a Waters 2D UPLC (Waters, United States) tandem with a Q Exactive high-resolution mass spectrometer (Thermo Fisher Scientific, United States) was used for the separation and detection of metabolites.

##### 2.5.2.1 Chromatographic Conditions

The column used was a CSH C18 column (1.7 μm, 2.1 × 100 mm, Waters, United States). Flow rate 0.35 ml/min, column temperature 55°C, injection volume 5 μl. Mobile phase in positive ionic mode: 10 mM ammonia formate, 0.1% formic acid and 60% acetonitrile in water (Liquid A) and 10 mM ammonia formate, 0.1% formic acid, 90% isopropanol and 10% acetonitrile in solution (Liquid B), mobile phase in negative ion mode: 10 mM ammonia formate and 60% acetonitrile in water (A solution) and 10 mM ammonia formate, 90% isopropanol and 10% acetonitrile (B solution). The elution conditions were carried out in a gradient: 0–2 min, 40%–43% B liquid; 2–2.1 min, 43%–50% B liquid; 2.1–7 min, 50%–54% B liquid; 7–7.1 min, 54%–70% B liquid; 7.1–13 min, 70%–99% B liquid; 13–13.1 min, 99%–40% B liquid, 13.1–15 min, 40% B liquid.

##### 2.5.2.2 Mass Spectrometry Conditions

Primary and secondary mass spectrometry data acquisition was performed using a Q Exactive mass spectrometer (Thermo Fisher Scientific, United States). Mass spectrometry scans the nucleus ratio range of 200–2,000, the first-class resolution is 70,000 and the AGC is 3e6, the maximum injection time is 100 ms. According to the precursor ion intensity, select Top3 for fragmentation, collect level information, the secondary resolution is 17,500, AGC is 1e5, maximum injection time is 50 ms, fragmentation energy is set to 15, 30, and 45 eV. Electron Spray Ionization (ESI) parameter settings: sheath gas flow rate is 40, the auxiliary gas flow rate is 10, spray voltage (|KV|) positive ion mode is 3.80, negative ion mode is 3.20, the ion transfer tube temperature is 320°C, and the auxiliary gas heater temp is 350°C.

When the instrument was tested, the samples were randomly ordered to provide more reliable experimental results, thus reducing systematic errors, and one QC sample was tested every 10 samples interspersed.

#### 2.5.3 Data Pre-Processing and Analysis

The raw UPLC-QTOF/MS data were exported through the MarkerLynx applications manager Version 4.1 (waters, United States) data processing system, and the spectra were subjected to peak extraction, peak alignment, peak filtering, and data modification to obtain metabolomics data. The data quality was evaluated using the repeatability of QC sample tests. The relative standard deviation (RSD) of the strength of the mass-to-charge ratio in the QC sample is less than 30% as a condition, and the poorly reproducible mass-to-charge ratio information in all samples is eliminated. The qualified data were imported into SIMCA-P14.1 statistical software (Umetrics, SE) for multivariate statistical analysis by partial least squares-discriminant analysis (PLS-DA) and those with variable importance in the projection (VIP) > 1 were screened as potential biomarkers (Pérez-Enciso and Tenenhaus, 2003; Gromski et al., 2015); and using univariate analysis of Fold Change and Student’s t-test methods, with VIP ≥ 1, Fold Change ≥ 1.2 or ≤ 0.83, *p* < 0.05 was used as a condition to screen different lipid metabolites between groups. Hierarchical Cluster was used for the selected differential lipid metabolites to show the changing trend of differential lipid metabolites between groups. The relevant metabolic pathways involved in the differential metabolites were analyzed by combining the Human Metabolite Database (HMDB database, http//www.hmdb.ca) and the Kyoto Encyclopedia of Genes and Genomes database (KEGG database, www.genome.jp/kegg/), as well as relevant literature reports.

### 2.6 The Gut Microbiota 16S Recombinant-DNA Amplification Sequencing

After the fecal samples were thawed, 180–200 mg were weighed for genomic extraction. The method was carried out by referring to the instructions of MagPure Stool DNA KF kit B (Magen, China). The extracted bacterial genomic DNA was quantified by Qubit fluorescence quantification and its quality was checked using 1% agarose gel electrophoresis.

Variable regions V3–V4 of bacterial 16S rDNA gene was amplified with degenerate PCR primers, 341F (5′-ACT​CCT​ACG​GGA​GGC​AGC​AG-3′) and 806R (5′-GGACTACHVGGGTWTCTAAT-3′). Both forward and reverse primers were tagged with Illumina adapter, pad, and linker sequences. PCR enrichment was performed in a 50 μL reaction containing a 30 ng template, fusion PCR primer, and PCR master mix. PCR cycling conditions were as follows: 94°C for 3 min, 30 cycles of 94°C for 30 s, 56°C for 45 s, 72°C for 45 s and final extension for 10 min at 72°C for 10 min.

The PCR products were purified with AmpureXP beads and eluted in the Elution buffer. Libraries were qualified by the Agilent 2100 bioanalyzer (Agilent, United States). The validated libraries were used for sequencing on the Illumina Hiseq 2500 platform (BGI, Shenzhen, China) following the standard pipelines of Illumina, and generating 2 × 300 bp paired-end reads. The off-machine data was filtered through data to filter out low-quality reads, and the remaining high-quality clean data can be used for post-analysis; Using the software FLASH (Fast Length Adjustment of Short reads, v1.2.11) (Magoč and Salzberg, 2011), paired reads obtained from double-end sequencing were assembled into one sequence using the overlapping relationship to obtain Tags of the highly variable region.

Then, operational taxonomic unit (OTU) representative sequences were taxonomically classified using Ribosomal Database Project (RDP) Classifier v.2.2 with a minimum confidence threshold of 0.6 and trained on the RDP database Release11.5 by QIIME v1.8.0. The USEARCH_global was used to compare all Tags back to OTU to get the OTU abundance statistics table of each sample. Alpha and beta diversity were estimated at the OTU level using MOTHUR (v1.31.2) and QIIME (v1.8.0), respectively (Schloss et al., 2009). Sample clustering was performed using the UPGMA-based QIIME (v1.8.0) software (Caporaso et al., 2010). Species accumulation curves were plotted using the R package version 3.1.1. Principal coordinate analysis (PCoA) was performed by QIIME (v1.8.0). Barplots were plotted at different taxonomic levels using Rpackagev 3.4.1. LEfSe clustering maps were drawn using the software LEfSe (https://huttenhower.sph.harvard.edu/galaxy/).

### 2.7 Association Analysis of Differential Gut Microbiota With Differential Lipid Metabolites

The data obtained for the differential lipid metabolites and the differential gut microbiota were reprocessed to obtain the relative abundance values for the co-expression clusters of differential lipid metabolites and the differential gut microbiota, respectively. Spearman rank correlation analysis and sparse generalized canonical correlation analysis were then used to correlate the two.

### 2.8 Data Statistics

The data were statistically processed using SPSS 23.0 software. The measurement data are expressed as mean ± standard error of means (SEM), and the count data were expressed as percentages (%). Differences between multiple groups were analyzed using one-way ANOVA for the measurement data, LSD-t-test as a post-hoc comparison if normal distribution and chi-squared were satisfied, otherwise, Dunnett-t-test was used, Kruskal-Wallis one-way ANOVA was used for the count data. *p* < 0.05 was considered a statistically significant difference. Graphs were plotted using GraphPad Prism 8.

## 3 Results

### 3.1 Apolipoprotein E-Deficient Mice Fed a Continuous High-Fat Diet Combined With Binding Stimulation for 16 weeks Gradually Developed Depression-Like Behavior

**FIGURE 1:**
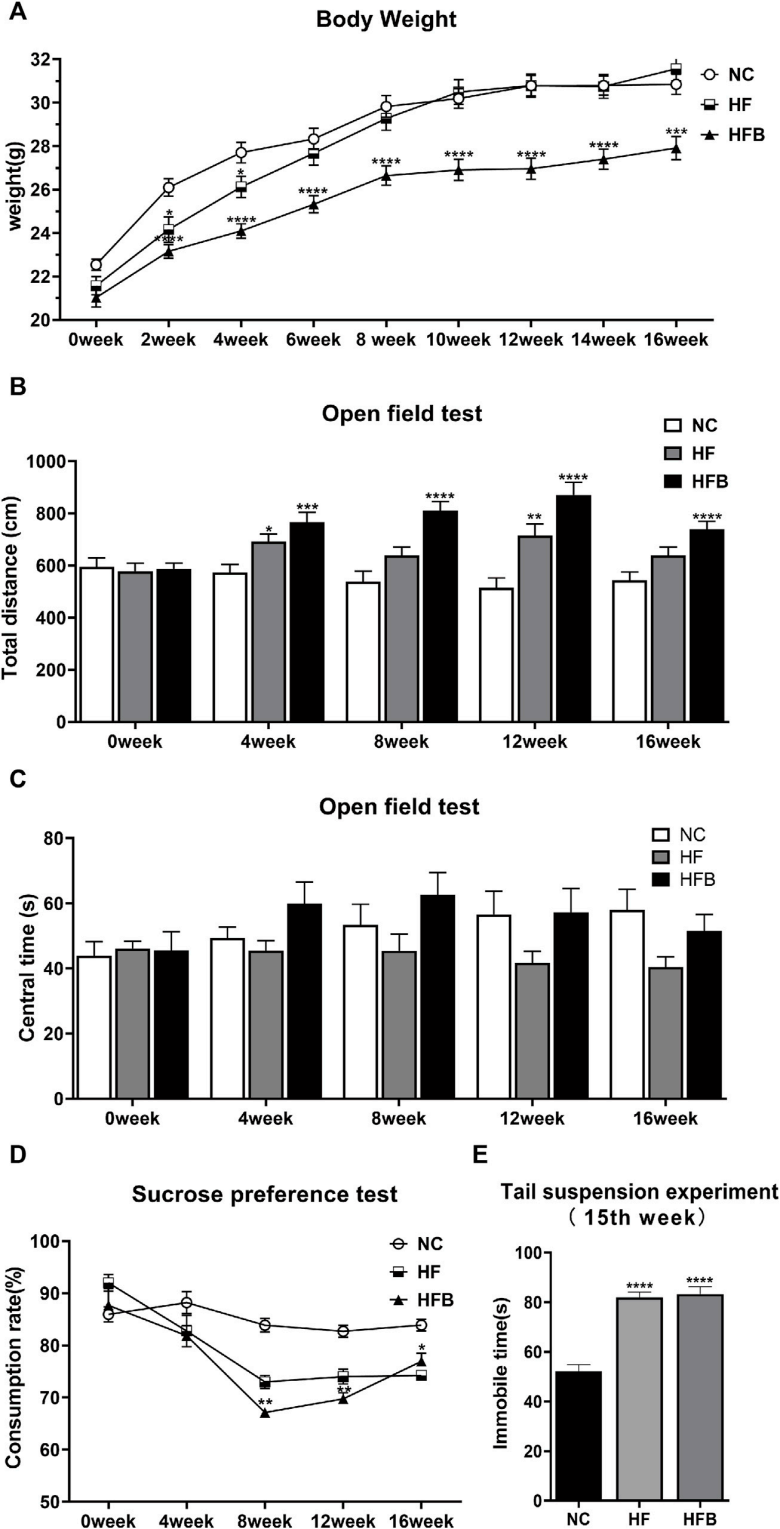
Behavioral changes in ApoE^−/−^ mice fed continuous high-fat combined with bound stimulation. **(A)** Measurement of body weight. **(B)** Total distance traveled in the open-field test. **(C)** The overall duration of stay in the central area of the open-field test. **(D)** Sucrose preference test. **(E)** Immobility time of the tail suspension experiment. *n* = 14, mean ± SEM. *, *p* < 0.05; **, *p* < 0.01; ***, *p* < 0.005; ****, *p* < 0.001; vs. NC.

We assessed whether the three groups of mice showed depression-like behavior by weighing, the sucrose preference test, the open field test, and the tail suspension test. As shown in Figure 1A, there was no significant difference in body weight between the groups at the beginning of the experiment. Over time, the mice in all three groups gained weight, but the mice in the HFB group gained weight more slowly. At second week, the mice in the HFB group had a statistically significant lower body weight compared to the NC and HF groups, and the difference persisted until the end of the experiment. This indicates that continuous binding stimulation had an inhibitory effect on the body weight gain of the mice and that the mice in the HFB group may have experienced a decrease in their ability to eat.

The open-field test was used to detect behavioral changes in the experimental animals by calculating the distance of spontaneous movement and the residence time in the central area of the open field. In terms of total horizontal movement distance (Figure 1B), the spontaneous movement distance increased significantly in the HF and HFB groups from the fourth week onwards, and the difference was statistically significant compared to the NC group. However, there was no significant difference between the three groups in terms of the overall duration of stay in the central area (Figure 1C). The analysis of the open-field experiments showed that the effect of a high-fat diet combined with binding stimulation increased the spontaneous motor capacity of ApoE^−/−^ mice, exhibiting behavioral characteristics similar to anxiety and hyperactivity.

The sucrose preference test is to determine the lack of pleasure by measuring the ratio of mice consuming sugar water. As shown in Figure 1D, at the beginning of the experiment, there was no significant difference in the rate of sugar water consumption among all groups. After the eighth week, the rate of sugar water consumption in the HFB group was significantly reduced compared to that in the NC group, and the difference was statistically significant. This indicates that the mice in the HFB group showed reduced preference for sweet solutions due to depressed mood and lack of pleasure, which is closely related to the behavior of depression.

The tail suspension test is used to assess the performance of desperate behavior of mice by measuring their Immobile time. As shown in Figure1E, 1 week before the end of the experiment, the mice in the HFB group spent significantly more time immobile compared to the mice in the NC group, and the difference was statistically significant. This indicates that the mice showed significant depressive behavior.

In summary, ApoE^−/−^ mice showed increased depression and anxiety behavior in comparison with ApoE^−/−^ mice fed only with a high-fat diet under the action of binding stimulation.

### 3.2 Apolipoprotein E-Deficient Mice had Increased Blood Lipids and the Formation of Atherosclerotic Plaques After 16 weeks of a High-Fat Diet

**FIGURE 2:**
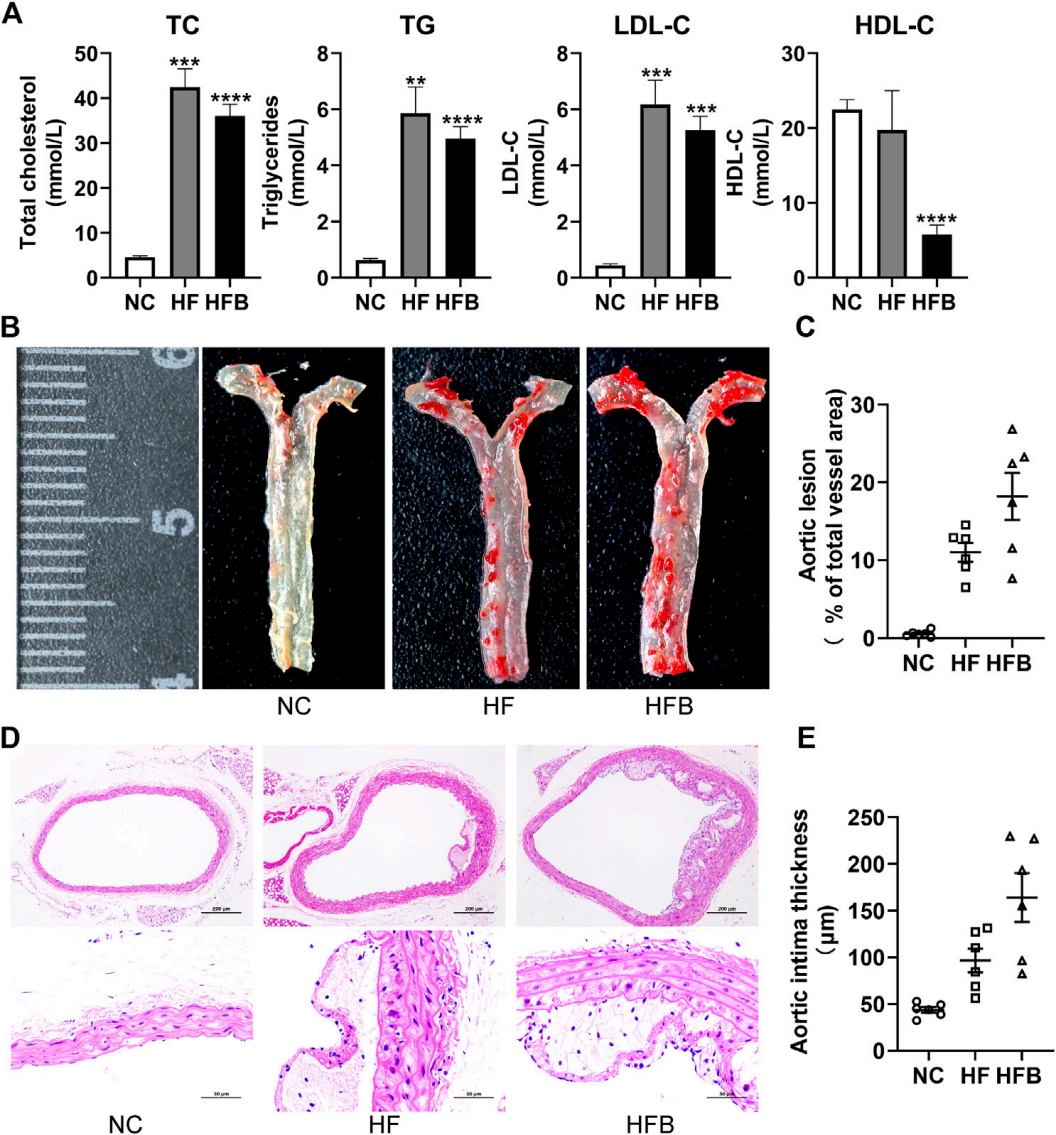
Evaluation of atherosclerosis formation in ApoE^−/−^ mice fed high-fat diets. **(A)** Plasma lipid levels in mice, from left to right: total cholesterol (TC), triglycerides (TG), low-density lipoprotein cholesterol (LDL-C), and high-density lipoprotein cholesterol (HDL-C), respectively. **(B)** Gross staining of oil red O in the thoracic aorta of mice (at the end of the 16th week). **(C)** Statistical map of thoracic aortic plaque area in mice. **(D)** HE staining of mice aortic arch (at the end of the 16th week; ×100, 100-fold; ×400, 400-fold). **(E)** Statistical maps of intima-media thickness (IT) in mice. *n* = 6, mean ± SEM. *, *p* < 0.05; **, *p* < 0.01; ***, *p* < 0.005; ****, *p* < 0.001; vs. NC.

As shown in Figure 2A, the serum levels of TC, TG, and LDL-C were significantly higher in the HFB and HF groups compared to the NC group, and the difference was statistically significant. HDL-C levels were significantly decreased in the HFB group compared to the NC group, and the difference was statistically significant. Interestingly, HDL-C levels were similar in the HF and NC groups.

As shown in Figure 2B, the inner wall of the thoracic aorta of C57BL/6 mice in the NC group was smooth without obvious red plaques; the inner wall of the aorta of mice in the HF and HFB groups had obvious scattered red plaques; the results of the quantitative analysis showed (Figure 2C), that the ratio of the atherosclerotic plaque area to the thoracic aortic vessel area in the HF and HFB groups showed a trend of gradual increase.

As shown in Figure 2D, the cross-sectional narrowing of blood vessels and the presence of large areas of lax tissue in the HF and HFB groups compared to the NC group mice were observed at 100-fold magnification views. In the 400-fold magnification views, the intima of the vessels in the HF and HFB groups were thickened, the endothelial cells and smooth muscle cells were disorganized, and atherosclerotic changes such as foam cell formation were seen.

Data acquisition of intima-media thickness was performed on HE-stained pathological images using Image-J software. As shown in Figure 2E, the intima-media thickness was gradually increased in mice in the HF and HFB groups compared to mice in the NC group, indicating thickened intima-media in mice aorta.

In summary, ApoE^−/−^ mice fed a high-fat diet for 16 weeks developed significant atherosclerotic plaques, successfully replicating the atherosclerotic model, and the symptoms became more severe after the addition of binding stimulation.

### 3.3 Lipidomic Analysis of the Prefrontal Cortex and Hippocampus Regions of Apolipoprotein E-Deficient Mice Fed a Continuous High-Fat Diet With Binding Stimulation

#### 3.3.1 Data Quality Control Analysis

**FIGURE 3:**
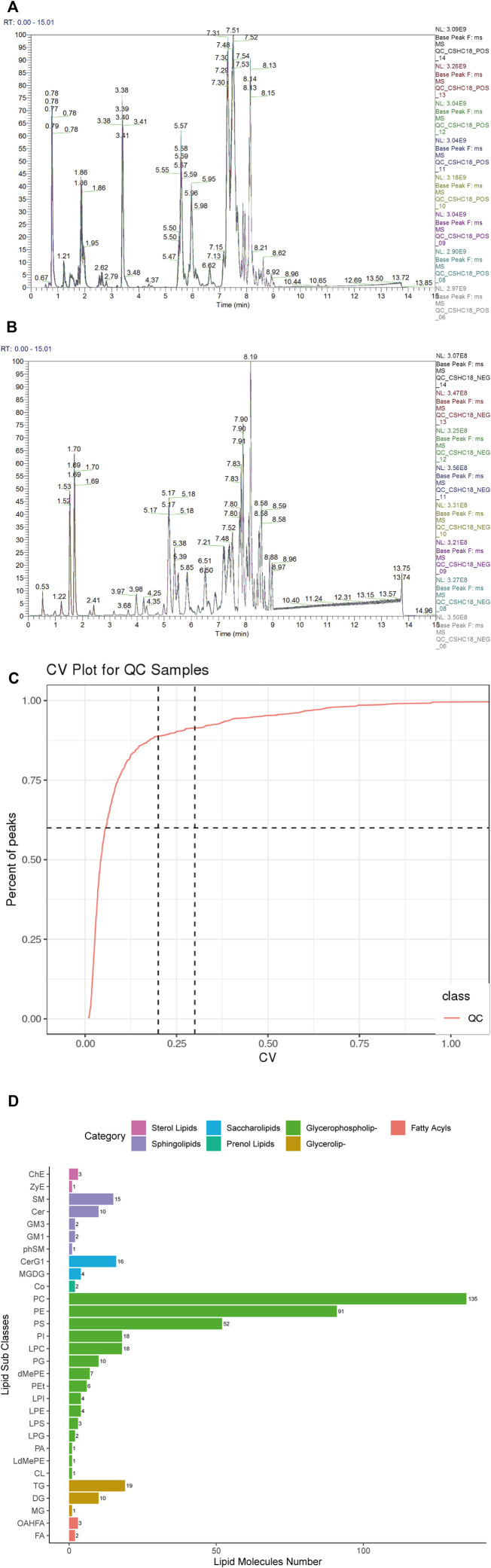
Quality control and lipid identification of lipidomic data from prefrontal cortex tissues of ApoE^−/−^ mice fed continuous high-fat diets combined with bound stimulation. **(A,B)** BPC (base peak chromatogram) overlays maps of QC samples from prefrontal cortex tissue (left: positive ion pattern; right: negative ion pattern). **(C)** CV distribution of lipid metabolites in QC samples from prefrontal cortex tissue. [RSD_30_number (RSD (CV) ≤30%): the number of lipid metabolites in QC sample with RSD (CV) less than or equal to 30%; RSD_Ratio: the ratio of the number of lipid metabolites with RSD (CV) less than or equal to 30% to the number of all detected lipid metabolites in QC sample; Ratio ≥ 0.6 means the data Ratio ≥ 0.6 is satisfactory] **(D)** Statistical plots of lipid subclasses and the number of corresponding lipid metabolites in prefrontal cortex tissue. The vertical axis shows the lipid subclasses identified in this experiment, the horizontal axis shows the number of lipid metabolites identified in each lipid subclass, different colors indicates different lipid categories.

Data quality is assessed by the reproducibility of QC sample assays. This included chromatogram overlap, the number of lipid metabolites, and differences in peak response intensity of QC samples. The BPC (base peak chromatogram) of all QC samples from the prefrontal cortex and hippocampus regions were overlaid, and the results from the prefrontal cortex regions (Figures 3A, B) and hippocampus regions (Supplementary Figures S1A, B) showed a good overlap of positive and negative ion spectra, with low fluctuations in retention time and peak response intensity, indicating that the instrument was in good condition and had a stable signal during the whole sample detection and analysis process. In the QC sample of prefrontal cortex tissue, the total number of lipid metabolites detected was 610 and the RSD_30_number was 557, as shown in Figure 3C, with an RSD_Ratio of 0.91 > 0.6. In the QC sample of hippocampus tissue, the total number of lipid metabolites detected was 713 and the RSD_30_number was 654, as shown in Supplementary Figure S1C shows that the RSD_Ratio was 0.92 greater than 0.6. indicating that the sequencing quality was all satisfactory.

#### 3.3.2 Detection and Identification of Lipid Metabolites in the Prefrontal Cortex and Hippocampus Regions of Apolipoprotein E-Deficient Mice Fed a Continuous High-Fat Diet With Binding Stimulation

After data pre-processing, a total of 444 lipid metabolites were detected in the prefrontal cortex regions of all samples, mainly in seven major classes and 30 subclasses, as shown in Figure 3D, and 532 lipid metabolites were detected in the hippocampus regions, mainly in seven major classes and 33 subclasses, as shown in Supplementary Figure S1D. The LipidSearch 4.1 software was then used to classify the lipid metabolites into four classes: A, B, C, and D. Classes A and B were identified at the Molecular Specie level, and classes C and D were identified at the Lipid Specie level. The results showed that there were 140 A classes, 81 B classes, 212 C classes, and 11 D classes in the prefrontal cortex regions and 179 A classes, 116 B classes, 224 C classes, and 13 D classes in the hippocampus regions.

#### 3.3.3 Analysis of Differential Lipid Metabolites in the Prefrontal Cortex and Hippocampus Regions of Apolipoprotein E-Deficient Mice Fed a Continuous High-Fat Diet With Binding Stimulation

To screen for differential lipid metabolites between groups, we used multivariate statistical analysis methods PLS-DA analysis and fold change analysis and Student’s t-test for analysis, respectively.

**FIGURE 4:**
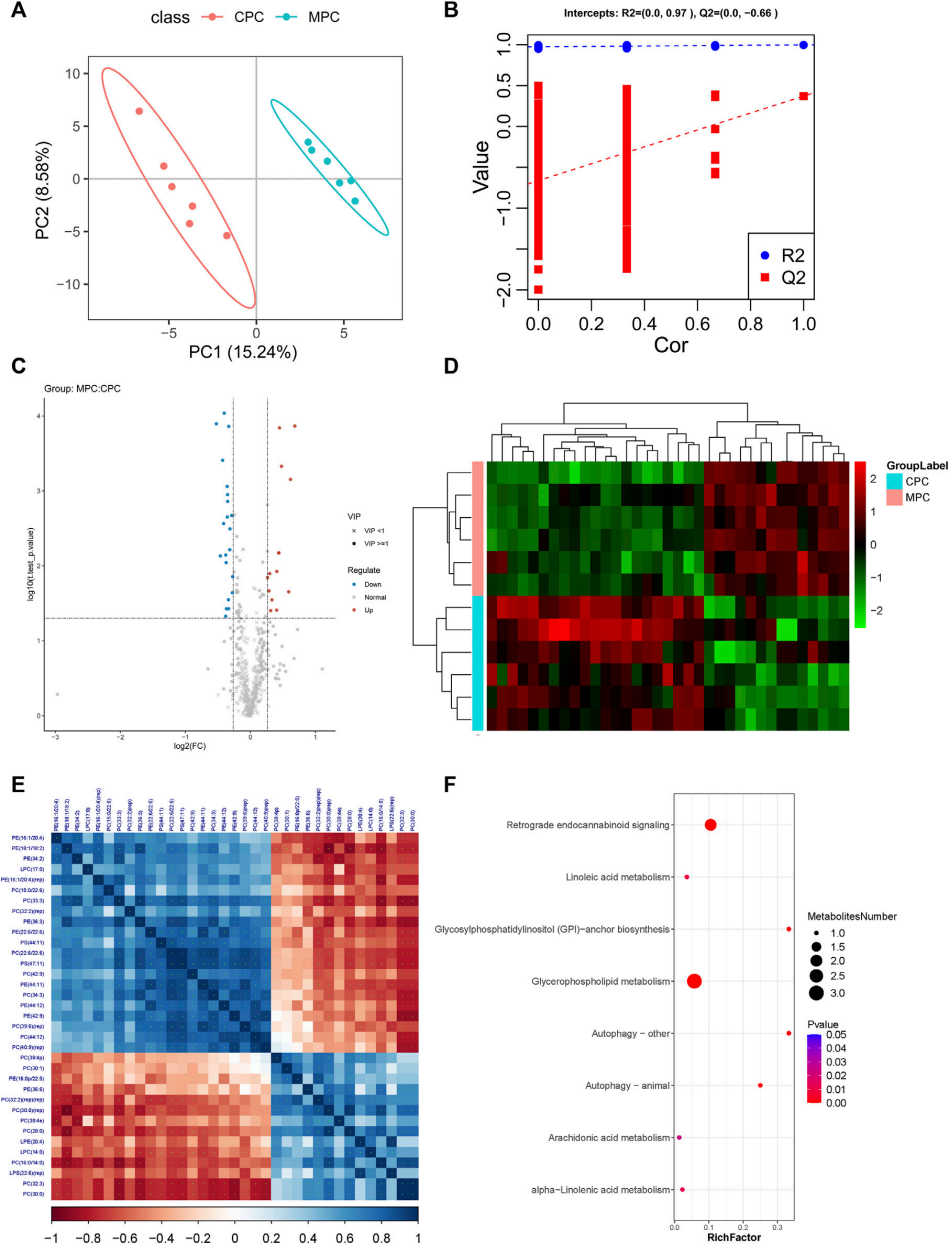
Molecular analysis of differential lipids in the prefrontal cortex tissues of ApoE^−/−^ mice fed a continuous high-fat diet combined with bound stimulation. CPC: Prefrontal cortex results of NC group, MPC: Prefrontal cortex results of HFB group. **(A)** Score plots for the PLS-DA discriminant analysis model of differential lipids in prefrontal cortex tissue. The horizontal axis is the first principal component and the vertical axis is the second principal component. Numbers in parentheses are the scores of that principal component, indicating the explanatory power of that principal component for the overall model. **(B)** Plots of response ranking tests for the PLS-DA analysis model of differential lipids in prefrontal cortex tissue. The two rightmost points in the plot are the true R2Y and Q2 values of the model, respectively, and the remaining points are the R2Y and Q2 values obtained from a random permutation of the samples used. **(C)** Volcano maps of differential lipids in prefrontal cortex tissue. Volcano maps were used to visualize the screened differential lipid metabolites. Blue is downregulated significant differential lipid metabolites, red are upregulated significant differential lipid metabolites, circles are lipid metabolites with VIP greater than or equal to 1, “×” are lipid metabolites with VIP less than 1, and non-significant lipid metabolites are grey. **(D)** The plot of differential lipid clustering analysis in prefrontal cortex tissue. Each column in the graph represents a differential ion, each row represents a sample, and different colors indicate different intensities, with colors ranging from green to red, indicating low to high intensity. **(E)** Heat map of molecular correlations of differential lipids in prefrontal cortex tissue, blue represents positive correlations, red represents negative correlations, the darker the color, the greater the linear correlation. “*” indicates a *p*-value < 0.05 for a statistical test of the correlation coefficient. **(F)** Enrichment map of KEGG metabolic pathway of differential lipid metabolites in prefrontal cortex tissue. The size of the dot represents the number of different lipid metabolites enriched in this pathway, the color represents the *p*-value, and the redder the color, the smaller the *p*-value.

PLS-DA analysis is the use of partial least squares regression to establish a model of the relationship between metabolite expression and sample category, which can reflect the difference between classification groups to the greatest extent. The VIP was also calculated to measure the strength and explanatory power of each metabolite expression pattern on the classification of each group of samples, thus aiding the screening of metabolic markers, which is generally considered to be greater than one to indicate a significant effect of the variable on the differentiation of sample categories. Data from prefrontal cortex tissue (Figure 4A; CPC: Prefrontal cortex results of NC group, MPC: Prefrontal cortex results of HFB group) and hippocampus tissue (Supplementary Figure S2A) were log2 logarithm transformation to create a PLS-DA model between the two sets of samples, the graph shows 15.24%–36.55% for PC1 and 8.58%–6.52% for PC2 respectively. The results show that the two groups of PLS-DA models of prefrontal cortex and hippocampus regions are separated, indicating that the lipid metabolites of the prefrontal cortex and hippocampus regions of the two groups of mice are different. Also, to evaluate the quality of the model building, we performed a 200 response permutation testing on the PLS-DA model. In general, the closer R2Y and Q2 are to one, the more stable and reliable the model is; Q2 is higher than 0.5, indicating that this model has a better prediction effect. The results showed that R2Y was 0.997 and Q2 was 0.372 in the prefrontal cortex regions (Figure 4B) and R2Y was 0.989 and Q2 was −0.03 in the hippocampus regions (Supplementary Figure S2B). (The lower Q2 may be due to the small number of samples and a large number of ions.)

Fold change analysis and t-test were performed on the data. Fold Change was obtained by Fold Change analysis, and the *p*-value pairs from the t-test were false discovery rate (FDR) corrected to obtain t.test_*p*.value_BHcorrect (*q*-value). The following conditions were defined for differential lipid metabolites screening: 1) VIP ≥ 1 for the first two principal components of the PLS-DA model; 2) Fold Change ≥ 1.2 or ≤ 0.83; 3) t.test_*p*.value < 0.05. The results of differential lipid metabolites obtained from prefrontal cortex and hippocampus regions screening in the HFB and NC groups were visualized by Volcano Plot visualized and displayed. As shown in Figure 4C, the number of differential lipid metabolites in the prefrontal cortex regions of the HFB and NC groups totaled 35, of which 14 were upregulated, including Lyso-phosphatidylcholine [LPC (14:0)], Lyso-phosphatidylserine [LPS (22:6) (rep)], lyso-phosphatidylethanolamine [LPE (20:4)], Phosphatidylcholine [PC (28:0), PC (30:1), PC (32:2) (rep) (rep), PC (16:0/14:0), PC (30:0) (rep), PC (30:0), PC (32:3), PC (38:4p), PC (38:4e)], Phosphatidylethanolamine [PE (36:6), PE (16:0p/22:5)], 21 were downgraded, including Lyso-phosphatidylcholine [LPC (17:0)], Phosphatidylcholine [PC (22:6/22:6), PC (44:12), PC (32:2) (rep), PC (15:0/22:6), PC (34:3), PC (42:9), PC (40:9) (rep), PC (39:6) (rep), PC (33:3)], Phosphatidylethanolamine [PE (22:6/22:6), PE (44:12), PE (44:11), PE (16:1/20:4), PE (16:1/20:4) (rep), PE (42:9), PE (34:2), PE (18:1/18:2)], PE (36:3), Phosphatidylserine [PS (44:11), PS (47:11)]. As shown in Supplementary Figure S2C, the number of differential lipid metabolites in the hippocampus region of the HFB and NC groups was 21 in total, with 10 upregulated, including Lyso-phosphatidylcholine [LPC (18:2), LPC (22:4)], Phosphatidylcholine PC (16:0/14:0), PC (22:4/20:4), PC (16:1/18:1), PC (16:0/20:3), Phosphatidylethanolamine [PE (18:0/22:5)], Phosphatidylserine [PS (41:6)], Phosphatidylethanolamine [PE (18:0/22:5)], Phosphatidylinositol [PI (20:4/20:4)], GD1a gangliosides1a [GD1a (d36:1)], 11 were downregulated, including phosphatidylcholine [PC (18:0/19:5), PC (33:3), PC (36:2) (rep)], Phosphatidylethanolamine [PE (22:5/22:6), PE (44:11) (rep), PE (37:5), PE (18:1/18:2), PE (36:3), PE (18:0p/20:1)], Phosphatidylserine [PS (17:0/18:1), PS (38:1)]. These differential lipid metabolites were then clustered using the Hierarchical Cluster method. As shown in Figure 4D and Supplementary Figure S2D, the differential lipid metabolites in prefrontal cortex and hippocampus regions tissue clustered relatively well between the NC and HFB groups, showing the emergence of variability between the two groups.

To understand the possible interactions between differential lipid metabolites, we performed correlation analyses of differential lipid metabolites in the prefrontal cortex and hippocampus tissue, respectively, to obtain Pearson correlation coefficients between different differential lipid metabolites and demonstrate the associations by plotting correlation heat maps. The results are shown in Figure 4E and Supplementary Figure S2E.

#### 3.3.4 Functional Annotation of the Differential Lipid Metabolites KEGG in the Prefrontal Cortex and Hippocampus Regions of Apolipoprotein E-Deficient Mice Fed a Continuous High-Fat Diet Combined With Binding Stimulation

We imported the differential lipid metabolites detected in the prefrontal cortex and hippocampus regions into the KEGG database for metabolic pathway enrichment analysis. The first eight pathways with significant differences and the highest degree of enrichment were screened out, and relevant functional enrichment maps were drawn. The pathway results of the main enrichment of differential lipid metabolites in the prefrontal cortex regions of the HFB group and the NC group are shown in Figure 4F, mainly the glycerophospholipid metabolism pathway (*p* = 4.17371e-07) and retrograde endocannabinoid signaling pathway (*p* = 1.497578e-05), the corresponding differential lipid metabolites are LPC (14:0), LPC (17:0), PC (15:0/22:6), PC (16:0/14:0), PE (16:1/20:4), PE (36:6), PC (15:0/22:6), PC (16:0/14:0), PE (16:1/20:4), PE (36:6). The results of the pathway of differential lipid metabolites enrichment in the hippocampus regions are shown in Supplementary Figure S2F, mainly the glycerophospholipid metabolism pathway (*p* = 2.091012e-07) and the retrograde endocannabinoid signaling pathway (*p* = 9.98998e-06), the corresponding differential lipid metabolites are: LPC (18:2), PC (16:0/14:0), PC (33:3), PE (18:0/22:5), PE (18:1/18:2), PC (16:0/14:0), PC (33:3), PE (18:0/22:5), PE (18:1/18:2).

### 3.4 Changes of the Gut Microbiota in Apolipoprotein E-Deficient Mice Fed Continuous High-Fat Diets Combined With Binding Stimulation

#### 3.4.1 16S Recombinant-DNA Sequencing Depth Data Reasonable

**FIGURE 5:**
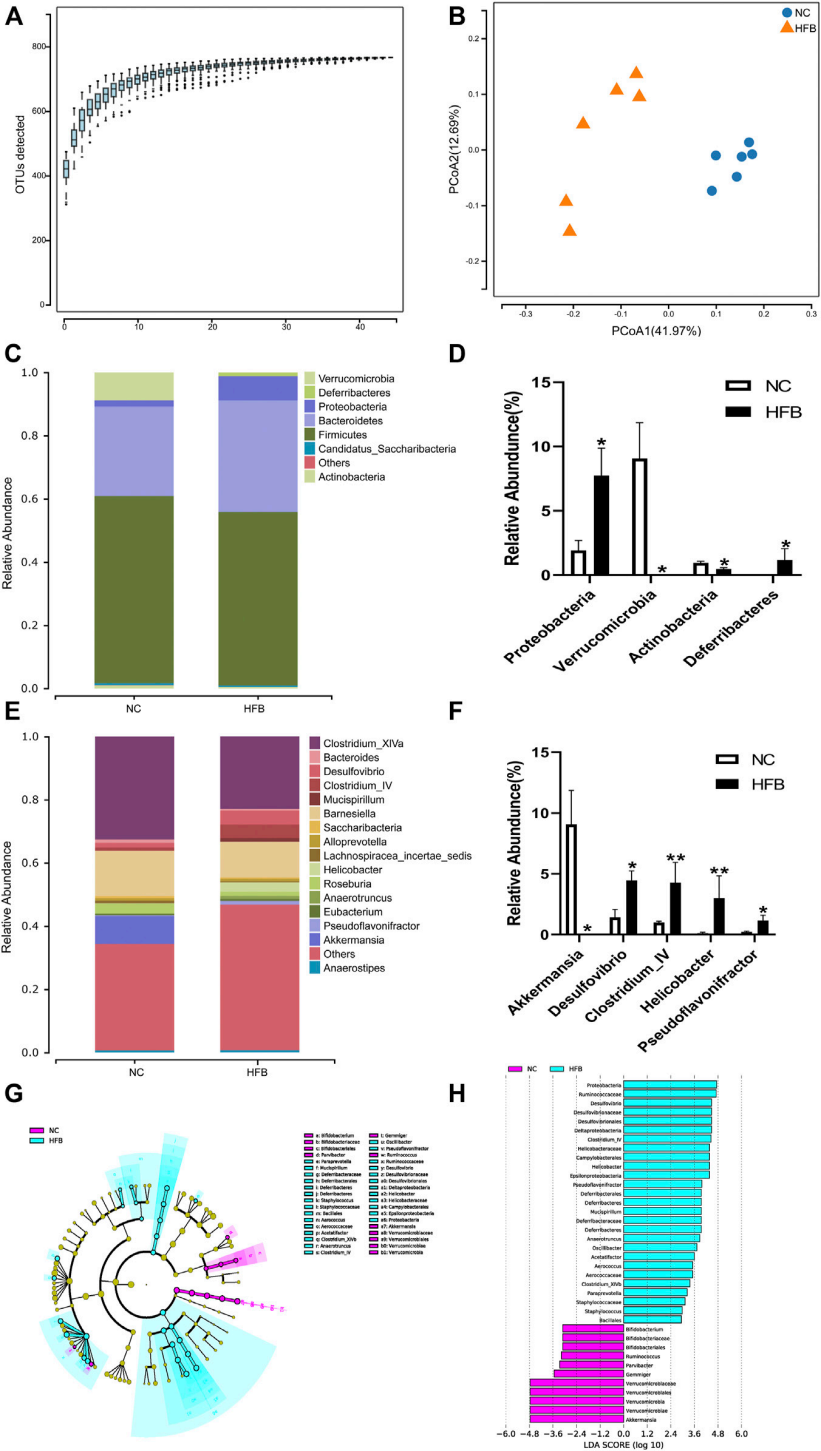
Changes in intestinal flora in ApoE^−/−^ mice fed continuous high-fat combined with bound stimulation. **(A)** Species accumulation graph. **(B)** PCoA plot. **(C,E)** Histograms of species composition [**(C)** phylum level; **E**: genus level]. **(D,F)** Statistical plots of differential species **(D)** phylum level; **(F)** genus level), mean ± SEM, *, *p* < 0.05; **, *p* < 0.01; vs. NC. **(G)** LEfSe clustering diagram, with different colors indicating different groups, and different colored nodes indicating microbial groups that play an important role in the group represented by the color, one color circle representing a biomarker, and yellow nodes indicating microbial groups that do not play an important role in the different groups. **(H)** LEfSe LDA plot.

Species accumulation curves are used to describe the increase in species with increasing sampling size and are a useful tool for understanding community species composition and predicting species richness, and are widely used in biodiversity and community surveys to determine the adequacy of sampling sizes and to estimate species richness. In the actual analysis, if the end of the curve still shows a sharp upward trend, it indicates that the sampling volume is insufficient, and new species can continue to be found by increasing the sample size; when the rising trend at the end of the curve tends to level off, it indicates that the sampling volume is sufficient. As shown in Figure 5A, the curves for each sample leveled off and the total number of OTUs in the community would no longer increase as new samples were added, indicating that the current sequencing depth was close to saturation for subsequent analysis.

#### 3.4.2 Changes in Colony Structure Distribution of the Gut Microbiota in Apolipoprotein E-Deficient Mice Fed Continuous High-Fat Diets Combined With Binding Stimulation

Fecal specimens from six mice in each group were sequenced to obtain valid sequences containing the tagged sequences, filtered for quality control, and spliced to obtain optimized sequences. We then annotated and analyzed the species of the gut microbiota using systematic taxonomic units (OTUs). To reduce data and overestimation of species diversity due to sequencing errors, OTUs at a 97% threshold level of sequence similarity were analyzed by clustering and then subjected to bioinformatic statistical analysis. Sequences were grouped to obtain the number of OTUs for each sample. The number of OTUs in each group is shown in Supplementary Figure S3A: 644 in the NC group and 602 in the HFB group. The same OTU was 521, 123 specific to the NC group and 81 specific to the HFB group. The results show that the number of OTUs decreased in the HFB group compared to the NC group.

Alpha diversity reflects the abundance and diversity of microbial communities within individual treatment group samples. We used the Sobs index to reflect the richness of each group’s community and the Shannon index to reflect the diversity of the community. The results are shown in Supplementary Figures S3B, C. Compared with the NC group, the Sobs and Shannon indices of the gut microbiota of mice in the HFB group were both reduced, indicating that the abundance and diversity of the gut microbiota of mice in the HFB group decreased.

Beta diversity is a comparative analysis of the microbial community composition of different samples. Given the differences in OTU abundance between the groups, we used the Weighted Unifrac calculation method for statistical purposes and the ggplot package of software: R (v3.4.1) to plot the differences in Beta diversity between groups. The results are shown in Supplementary Figure S3D. The Beta diversity values in the HFB group were significantly lower compared to the NC group (*p* = 0.036), indicating that the community structure of the mice’s gut microbiota changed significantly in the atherosclerosis co-depression disease state.

PCoA (Principal Co-ordinates Analysis) analysis, which can show the similarity or difference of data, is a non-binding data dimensionality reduction analysis method that can be used to explain the differences in the structure of the gut microbiota of each group of mice, the lower the coincidence rate between groups and the farther apart, it proves that the composition of the intestinal microbiota of the two groups of mice is different. The final PCoA display map was obtained by using the software: QIIME (v1.80), using an iterative algorithm, using 75% of the sequence count of the sample with the lowest sequence count of all samples for sampling analysis, and combining the statistics after 100 iterations. As shown in Figure 5B, the NC group was mainly distributed in the second and third quadrants, expressing 12.69% of the total variability, while the gut microbiota of mice in the HFB group was mainly concentrated in the fourth quadrant, explaining 41.97% of the total variability. The separation between the two groups was significant and the difference was statistically significant (*p* = 0.002), demonstrating a greater difference in the composition of the gut microbiota between the NC and HFB groups.

#### 3.4.3 Species Composition and Difference Species Analysis of Gut Microbiota at Different Taxonomic Levels Between Groups

In this study, the abundance composition and differential species of the two groups of mice were analyzed at the phylum level and genus level respectively.

At the phylum level, as shown in Figure 5C, the most dominant microbial groups were *Firmicutes*, *Bacteroidetes*, *Proteobacteria*. we show this by selecting the top 10 species in abundance and selecting those with significant differences between groups, as shown in Figure 5D, which shows that, compared to the NC group, the gut microbiota of mice in the HFB group had *Proteobacteria* (*p* = 0.013), Deferribacteres (*p* = 0.013) had a statistically significant increase in abundance; *Verrucomicrobia* (*p* = 0.019), *Actinobacteria* (*p* = 0.020) had a statistically significant decrease in abundance compared to the NC group. The study showed that the ratio of *Actinobacteria*/thick-walled bacteria has been considered as one of the markers of human health and that the ratio of *Actinobacteria*/thick-walled bacteria was significantly increased in the HFB group compared to the NC group.

At the genus level, as in Figure 5E, the main microbial taxa were *Clostridium_XlVa*, *Barnesiella*, and *Akkermansia*. species with significant differences are shown in Figure 5F, where compared to the NC group, the HFB group *Desulfovibrio* (*p* = 0.020), *Clostridium_IV* (*p* = 0.005), *Helicobacter* (*p* = 0.008), and *Pseudoflavonifractor* (*p* = 0.020) increased in abundance with statistically significant differences; *Akkermansia* (*p* = 0.019) decreased with statistical significance.

To further identify communities or species that produced significant differential effects, we used LEfSe analysis to assess the magnitude of the effect of each species’ abundance on the differential effect, and the results are shown in Figures 5G, H, which only shows the difference indicator species that meet the LDA (linear discriminant analysis) significance threshold greater than 2.0. The results show that the bacteria group that plays a significant role in the feces of the NC group mice is *Proteobacteria*, *Ruminococcaceae*, and The gut microbiota that play a significant role in the feces of mice in the HFB group are *Verrucomicrobiaceae*, *Verrucomicrobiales*, *Verrucomicrobia*, *Verrucomicrobiae*, *Akkermansia*.

### 3.5 Association Analysis of Differential the Gut Microbiota and Associated Brain Region Differential Lipid Metabolites in Apolipoprotein E-Deficient Mice Fed Continuous High-Fat Diets Combined With Binding Stimulation

To further explore the relationship between differential gut microbiota and differential lipid metabolites, we used Gene Co-expression Network Analysis (WGCNA) to downscale the differential lipid metabolome data from the prefrontal cortex and hippocampus regions to obtain co-expression clusters of differential lipid metabolites, and then used Spearman rank correlation analysis and sparse generalized canonical correlation analysis were used to correlate them with the relative abundance of differential clusters at the phylum level.

**FIGURE 6:**
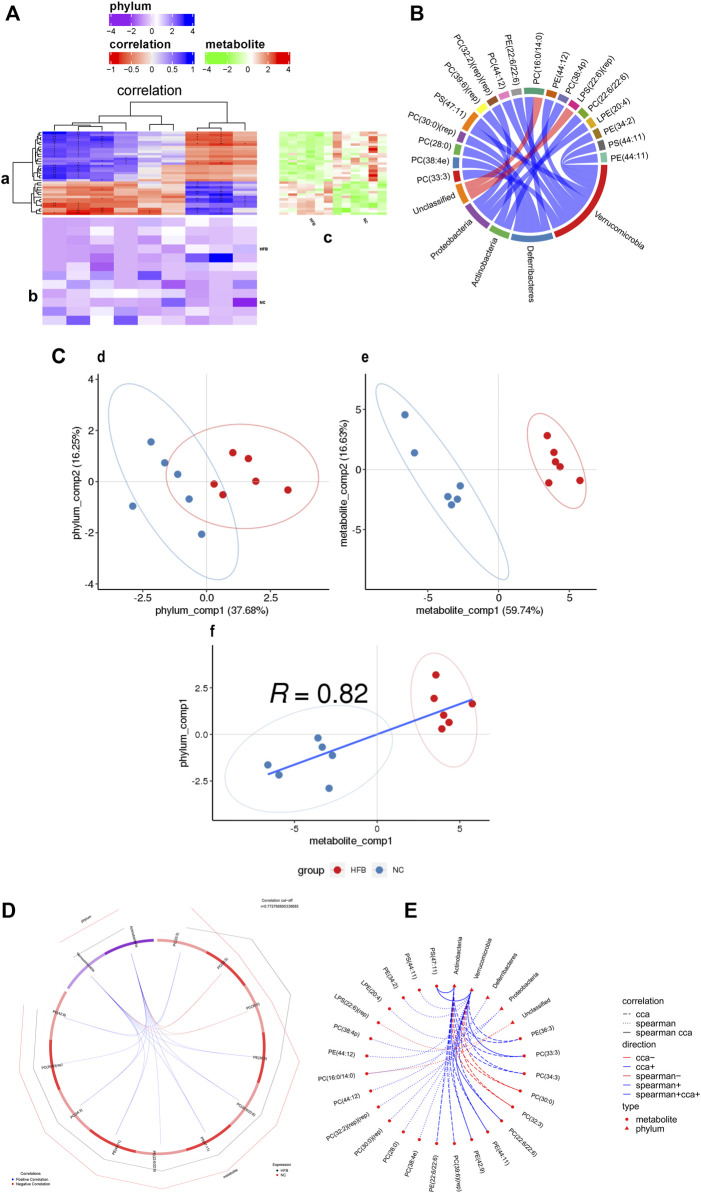
Correlation analysis of differential metabolites and microbial taxa in the prefrontal cortex tissue of ApoE^−/−^ mice fed continuous high-fat diets combined with bound stimulation. **(A)** Heat map of the correlation between differential metabolites and microbial taxa in prefrontal cortex tissue. **(a)** clustering heat map of correlation between differential metabolites and microbial taxa, horizontal coordinates are microbial taxa, vertical coordinates are differential metabolites, * indicates *p* value < 0.05,** indicates *p* value < 0.01; red indicates a negative correlation, blue indicates positive correlation, the darker the color, the stronger the correlation; **(b)** heat map of relative abundance of microbial taxa, rows represent samples, row names are group names, columns represent microbial taxa, those with column names are differential microbial taxa, from yellow to red indicate low to high relative abundance. **(c)** heat map of differential metabolite abundance, rows represent differential metabolites, columns represent samples, column names are group names; Pathway: metabolic pathways significantly enriched in differential metabolites, each color other than grey represents a metabolic pathway. The complex heat map is clustered based on the correlation coefficients in plot **(a)**. Microbial taxa with high correlation with differential metabolites are clustered together in plot **(b)**, while differential metabolites with high correlation with microbial taxa are clustered together in plot **(C)**. **(B)** Chord diagram of the correlation between differential metabolites and microbial taxa in prefrontal cortex tissue. Each node represents a differential metabolite or microbial taxon, and the red arcs between nodes represent negative correlations and the blue arcs represent positive correlations. **(C)** Scatter plot of the correlations between differential metabolites of prefrontal cortex tissue and microbial taxa. **(d)** scatter plot of microbial taxon components, the horizontal coordinate is the first component value, the vertical coordinate is the second component value; **(e)** scatter plot of differential metabolite components, the horizontal coordinate is the first component value, the vertical coordinate is the second component value; **(f)** scatter plot of Pearson correlation between differential metabolites and microbial taxon first components, the horizontal coordinate is the metabolic pathway first component value, the vertical coordinate is the microbial taxon first component value. A larger R indicates a higher degree of correlation between microbial taxa and the first component of the metabolic pathway. **(d,e,f)** Each point in the plot represents a sample, and the colors and ovals represent sample groupings; the greater the sample dispersion in different groups, the better the classification of that component value. **(D)** Ring plots of differential metabolite and microbial correlations in prefrontal cortex tissue. The broken line on the periphery of the ring represents the abundance value of the differential metabolite and the microbial group in each group, the distance between the broken lines represents the difference between the groups, the upper right corner is the correlation coefficient threshold, and only the differential metabolites with the absolute value of the correlation coefficient greater than the threshold There is a connection with the microbial group. The blue connection represents a positive correlation, and the red connection represents a negative correlation. **(E)** Network diagram of a correlation between differential metabolites and microbial taxa in prefrontal cortex tissue. Circles are metabolites, triangles are microbial taxa, red lines indicate negative correlations, blue lines indicate positive correlations, long dashed lines indicate typical correlations, short dashed lines indicate rank correlations and solid lines indicate typically and rank correlations.

First, the correlation between differential lipid metabolites and differential gut microbiota was analyzed by calculating the rank correlation coefficients between the two. As shown in Figure 6A and Supplementary Figure S4A, the difference in lipid metabolites in the prefrontal cortex and hippocampus regions of the two groups are clustered heat maps of the correlation between the difference in phylum level and the gut microbiota, the results demonstrate the correlation that exists between differential metabolites and gut microbiota as well as the metabolic pathways in which differential metabolites are significantly enriched. We selected the top 20 pairs of relationships with the strongest rank correlation (largest absolute value of correlation coefficient and *P* value < 0.05) to display as chordal plots, as in Figure 6B and Supplementary Figure S4B. prefrontal cortex regions differential lipid metabolites PC (33:3), PC (39:6) (rep), PC (44:12), PE (22:6/22:6), PE (44:12), PS (47: 11), PE (34:2), PS (44:11), PE (44:11) were positively correlated with *Verrucomicrobia*; PS (47:11), PC (22:6/22:6) were positively correlated with *Actinobacteria*; PC (38:4e), PC (28:0), PC (30:0) (rep), PC (16:0/14:0) were positively correlated with *Deferribacteres*; PC (32:2) (rep) (rep), PC (38:4p), LPE (20:4) were positively correlated with *Proteobacteria*. The differential lipid metabolites PC (33:3) and PE (36:3) in the hippocampus regions were positively correlated with *Actinobacteria*; PI (20:4/20:4), PS (41:6), PC (22:4/20:4), PC (16:0/20:3), LPC (22:4) were negatively correlated with *Actinobacteria*; PC (16:0/20:3) was negatively correlated with *Verrucomicrobia*; PE (37:5), PC (18:0/19:5) was negatively correlated with *Deferribacteres*; PC (16:0/14:0), PC (16:1/18:1) was positively correlated with *Deferribacteres*; PE (37:5), PC (18:0/19:5) were negatively correlated with *Proteobacteria*; PI (20:4/20:4) were positively correlated with *Proteobacteria*.

Typical correlation analysis is a multivariate dimensionality reduction analysis method that enables the integration of differential metabolite and differential gut microbiota datasets and can be used to identify highly correlated and taxonomically effective biomarkers. Figure 6C and Supplementary Figure S4C show scatter plots of the values of differential lipid metabolites and their differential gut microbiota components at the prefrontal cortex and hippocampus regions groups, respectively. The results show the effect of sample classification for the first and second component values of differential lipid metabolites and differential gut microbiota, with a large degree of sample dispersion between the HFB and NC groups, indicating a good classification effect. It also shows that the correlation R values between the first component of the differential lipid metabolite and the first component of the differential gut microbiota are 0.82 and 0.62, respectively, It shows that the difference in lipid metabolites between the HFB group and the NC group has a high degree of correlation with the first component of the different gut microbiota. Subsequently, we selected the top 20 pairs of relationships that took the strongest typical correlations (largest absolute values of correlation coefficients) to display as a loop, as shown in Figure 6D and Supplementary Figure S4D. The differential lipid metabolites in the prefrontal cortex regions PC (22:6/22:6), PS (47:11), PE (36:3), PC (33:3), PC (34:3), PE (44:11), PE (42:9), PC (39:6) (rep), PE (22:6/22:6) were positively correlated with *Actinobacteria*; PC (30:0), PC (32:3) were negatively correlated with *Actinobacteria*; PC (22:6/22:6), PS (47:11), PC (34:3), PE (36:3), PC (33:3), PE (42:9), PE (44:11) were positively correlated with *Verrucomicrobia*; PC (30:0), PC (32:3) were negatively correlated with *Verrucomicrobia*; differential lipid metabolites in the hippocampus regions PE (18:1/18:2), PE (18:0p/20:1), PE (36:3), PC (33:3), PE (22:5/22:6), PE (44:11) (rep) were positively correlated with *Actinobacteria*; PE (18:0/22:5), PC (16:1/18:1), PS (41:6), LPC (22:4), PC (16:0/14:0) were negatively correlated with *Actinobacteria* were negatively correlated; PE (18:0/22:5), PC (16:1/18:1), PS (41:6) were positively correlated with *Proteobacteria*; PE (18:0p/20:1), PE (18:1/18:2), PE (36:3), PC (33:3) were negatively correlated with *Proteobacteria* was negatively correlated; PE (36:3) was positively correlated with *Verrucomicrobia*.

Finally, we combined the top 20 relationship pairs with the strongest previous rank correlations and the top 20 pairs with the strongest typical correlations to plot a correlation network, as shown in Figure 6E and Supplementary Figure S4E suggesting that the above-screened differential gut microbiota were strongly correlated with differential lipid metabolites.

## 4 Discussion

With the transformation of the modern medical model to the bio-psycho-social model, psycho-cardiology has attracted more and more attention (Xue et al., 2020). During diagnosis and treatment, patients with coronary heart disease are often accompanied by persistent chronic stress response stimuli, such as stress, pain, and other physiological factors, as well as psychological factors brought by family environment and social environment, which increase their susceptibility to depression (Siegrist and Sies, 2017). To better simulate the living conditions of patients with coronary heart disease or atherosclerosis, this study used 16 weeks of high-fat diet combined with binding stimulation to induce the classic atherosclerosis model ApoE^−/−^ mice cardiovascular pathological changes and depression-like mood to establish the atherosclerosis co-depression mice model. First, the depression-related behavioral results of body weight, sucrose preference test, open field test, and tail suspension test showed that the depression state of the HFB group was gradually worse than that of the NC group, showing the characteristics of depression and anxiety. At the same time, ApoE^−/−^ mice fed with high-fat diets showed abnormal lipid metabolism due to the functional inactivation of the *ApoE* gene, which was manifested by increased lipid indexes in the serum and significant plaque formation and lipid deposition in the inner wall of the aorta. These results suggest that this study successfully replicated the mouse model of atherosclerosis co-depression. In addition, we found that binding stimulation made symptoms of atherosclerosis more severe in ApoE^−/−^ mice.

At present, studies on the mechanism of the cardiovascular disease combined with psychological disease mainly focus on inflammation, neuroendocrine system, genes, and social behavior, and is explained with the help of genomics, transcriptomics, and proteomics (Dhakan et al., 2019). However, we also realize that gene changes may not be necessarily expressed, and the metabolites are the final results of a series of changes in the body, which can more accurately reflect the pathological and physiological state of the body. In particular, abnormal lipid metabolism is considered to be one of the important factors that promote the occurrence and development of atherosclerosis plaques (Kurilshikov et al., 2019), indicating that it is necessary to explore the process of lipid metabolism in the body. With the development of depression, changes in brain structure and function occur, especially damage to the prefrontal cortex and hippocampus regions, leading to mood disorders and cognitive impairment of memory, attention, and executive function (Li et al., 2019). In this study, we used the LC-MS/MS technique for non-targeted lipidomics analysis, combined with multi-dimensional and one-dimensional statistical analysis, and found the differences in lipid metabolites in the prefrontal cortex and hippocampus regions of atherosclerosis co-depression mice and normal mice. Interestingly, we compared these differential lipid metabolites in the KEGG database. It was found that the most influential metabolic pathways in the prefrontal cortex and hippocampus regions were the glycerophospholipid metabolism pathway and the retrograde endocannabinoid signaling pathway.

The glycerophospholipid metabolism pathway and the retrograde endocannabinoid signaling pathway have long been shown to regulate depression in the brain. A depression model with cynomolgus macaques as the research object proved that disorders of hippocampus glycerophospholipid metabolism pathway in cynomolgus macaques were a marker of depression (Zheng et al., 2020). The abnormality of the glycerophospholipid metabolism pathway is mainly manifested as an abnormality of cell membrane synthesis. Glycerophospholipids are critical components of neuronal membranes and myelin and principal regulators of synaptic function. They determined the location and function of various receptors. Compared with other tissues, the brain is particularly rich in lipid compositions and content (Bozek et al., 2015). Studies have shown that the membrane-forming lipids in the brain play a vital role in depression (Adibhatla and Hatcher, 2008). The nerve cell membranes of the prefrontal cortex and hippocampus regions are composed of a large number of lipids. These lipid metabolites can directly control the assembly of signal proteins on the membrane, and thus have important effects on nerve function and signal transduction. When the lipid metabolism on the neuronal membrane is abnormal, the nerve cell function in the prefrontal cortex and hippocampus regions will be abnormal, for example, the activation of neuroinflammatory pathways in the prefrontal cortex and hippocampus regions will lead to depression-like cognitive dysfunction (Lee et al., 2018). In addition, studies have found that depression is also associated with decreased expression of enzymes related to the glycerophospholipid metabolism pathway (Zhang et al., 2018b). Endocannabinoids (eCBs) are a family of lipid molecules that act as key regulators of synaptic transmission and plasticity (Araque et al., 2017). The retrograde endocannabinoid signaling pathway is the principal mode by which endocannabinoids mediate short-term and long-term forms of plasticity at both excitatory and inhibitory synapses (Castillo et al., 2012). 2-arachidonoylglycerol (2-AG), one of the endocannabinoids, produced from membrane lipids upon postsynaptic Ca2D elevation and/or activation of Gq/11-coupled receptors, and released from postsynaptic neurons. The released 2-AG then acts retrogradely onto presynaptic cannabinoid CB1 receptors and induces suppression of neurotransmitter release either transiently or persistently (Kano, 2014). It is widely believed that eCBs regulate a wide range of neurological functions, including cognition, motor control, feeding behavior, and pain by regulating the strength of synapses. In this study, the main differential lipid metabolites enriched in the glycerophospholipid metabolism pathway and the retrograde endocannabinoid signaling pathway were glycerophospholipids. The value includes LPC, PC, and PE. Therefore, we can conclude that the depression-like behavior in the HFB group mice may be related to the disorder of the glycerophospholipid metabolism pathway and the retrograde endocannabinoid signaling pathway, which leads to abnormal white matter in the prefrontal cortex and hippocampus regions, and synaptic dysfunction.

The gut microbiota is closely related to human health and is called a special “organ” of the human body. The gut microbiota obtains nutrients and energy from the gut of the host, assists the host in digestion and absorption of nutrients, and regulates and balances the immune and metabolic functions of the host (Romaní-Pérez et al., 2021). In recent years, there has been a significant increase in the study of the gut microbiota in cardiovascular disease and depression, but less in atherosclerosis co-depression. Studies have shown that the gut microbiota disorders and their metabolites TMAO, Bas, and SCFAs can cause abnormal lipid metabolism and lipid deposition in the host, causing inflammation, which is closely related to atherosclerosis (Lindskog Jonsson et al., 2018; Wu et al., 2020; Zhang et al., 2021). In addition, the gut microbiota can also affect brain function and behavior through the “microbiota-gut-brain” axis, leading to the occurrence of depression (Seguella et al., 2021). Animal experiments have shown that changes in the gut microbiota can induce anxiety and depression behavior through changes in an inflammatory response, hypothalamus-pituitary-adrenal axis (HPA), or neurotransmitter signal transmission (Jiang et al., 2015). Therefore, the differential changes in the structure, functional genes, and metabolic activity of the gut microbiota may be the underlying mechanism of atherosclerosis co-depression. Then, in this study, whether there is any difference in the gut microbiota of ApoE^−/−^ mice fed with continuous high-fat diet combined with binding stimulation and C57BL/6 mice fed with normal diet only, and is there any correlation with the differential lipid metabolisms screened above?

In previous studies, the abundance of *Firmicutes* and *Bacteroidetes* in the gut microbiota of depressed patients or mice were mostly different. In this study, although *Firmicutes* and *Bacteroidetes* were the dominant phyla of species abundance of mice in the HFB group, there were no significant differences compared with mice in the NC group, while other bacteria showed differences, which may be related to the degree of depression or sample size of mice in HFB group. A large number of studies have demonstrated that depression is often accompanied by immune activation, which is characterized by increased secretion of pro-inflammatory cytokines (Hayley et al., 2021). Moreover, immune activation can also cause changes in individual mood and behavior (Zhu et al., 2019). *Desulfovibrio* is a Gram-negative bacteria, and its increased abundance in the intestines will result in high levels of lipopolysaccharide (LPS), which will damage the gut barrier function (Zhang et al., 2018a). LPS is an immune activator and cytokine inducer that has been used in many studies related to inflammation. Peripheral injection of LPS can activate the innate immune system through toll-like receptor four and promptly induce the release of some pro-inflammatory cytokines, such as interleukin (IL)-1β, TNF-α, and IL-6. Studies have shown that the LPS depression model induced immune stress by injection of LPS obtained from *Escherichia coli* leading to depression-like behavior in mice (Chung et al., 2008). *Akkermansia*, as a probiotic, is located in the mucous layer of the gut tract and acts as a barrier against exogenous pathogens (Zhang et al., 2019). It produces a metabolite called nicotinamide (NAM) to reduce diet-induced oxidative stress and inflammation and accelerate glucose metabolism. Currently, *Akkermansia* has been shown to modulate metabolic endotoxemia, inflammation, and gut barrier integrity (Cani, 2018; Wang et al., 2020). In this study, we found that the depression-like behavior of atherosclerosis mice may be related to the changes in the gut microbiota caused by long-term high-fat diet combined with binding stimulation, especially the increase of *Desulfovibrio* abundance and the decrease of *Akkermansia* abundance. This may be associated with increased gut inflammation and decreased gut permeability, leading to the release of inflammatory cytokines. *Desulfovibrio* and *Akkermansia* are expected to be potential biomarkers and therapeutic targets for atherosclerosis co-depression (Zhu et al., 2018).

To further understand the mechanisms underlying the gut microbiota on atherosclerosis co-depression and the “microbiota-gut-brain” axis, it is necessary to explore the relationship between the gut microbiota and lipid metabolism in the prefrontal cortex and hippocampus regions. In this study, association analysis of the data showed that the differential lipid molecules in the prefrontal cortex and hippocampus regions of atherosclerosis co-depression mice and normal mice were strongly correlated with the differences in gut microbiota at the phylum level. Disorders of the glycerophospholipid metabolism pathway and the retrograde endocannabinoid signaling pathway in the prefrontal cortex and hippocampus regions of mice with atherosclerosis co-depression were demonstrated to be related to the decrease of gut permeability and the release of inflammatory factors caused by changes in gut microbiota.

In conclusion, after 16 weeks of continuous high-fat diet combined with binding stimulation, ApoE^−/−^ mice showed symptoms of atherosclerosis co-depression. The mechanism may be the disorder of lipid metabolism pathway in the prefrontal cortex and hippocampus regions, and the change of gut microbiota in ApoE^−/−^ mice, especially the influence of *Desulfovibrio* and *Akkermansia*. In addition, we further confirmed the existence of the “microbiota-gut-brain axis” through association analysis, and confirmed that the disorder of the lipid metabolism pathways in the prefrontal cortex and hippocampus regions was closely related to the gut microbiota in atherosclerosis co-depression mice.

## Data Availability

The metabolomics data contained in the article has been stored in the EMBL-EBI MetaboLights database (DOI: 10.1093/nar/gkz1019, PMID: 31619833), and the identifier is MTBLS3596. The 16s rDNA sequencing data contained in the article have been uploaded to NCBI (accession number: PRJNA770133).

## Abbreviations

ApoE^−/−^, apolipoprotein E-deficient; 16S rDNA, 16S recombinant-DNA; NC, normal control; HF, hyperlipid feeding; HFB, hyperlipid feeding combined with binding; QC, quality control; RSD, relative standard deviation; PLS-DA, partial least squares-discriminant analysis; VIP, variable importance in the projection; KEGG database, Kyoto Encyclopedia of genes and Genomes database; OTU, operational taxonomic unit; PCoA, principal coordinate analysis.
