# Supplementary material for: Effects of the Lipid Metabolites and the Gut Microbiota in ApoE−/− Mice on Atherosclerosis Co-Depression From the Microbiota-Gut-Brain Axis
Source: Front Mol Biosci. 2022 Apr 26;9:786492. doi: 10.3389/fmolb.2022.786492 (PMC9086493; doi:10.3389/fmolb.2022.786492)
Supplement: Supplementary file 1 [file DataSheet1.docx]

*Supplemental Material*

**1 Supplementary Tables**

**Animals**

| **Species** | **Vendor or Source** | **Background Strain** | **Sex** | **Persistent ID / URL** |
| --- | --- | --- | --- | --- |
| Mice | Beijing Weishang Lituo Technology Co., Ltd. | ApoE^-/-^; C57BL/6 | ♂ | 110364200100061018 |

**Equipment or reagent**

| **Description** | **Source / Repository** | **Persistent ID / URL** |
| --- | --- | --- |
| Oil red staining solution | Servicebio | G1016 |
| Fixative | Servicebio | G1101 |
| Isopropyl alcohol | Sinopharm Chemical Reagent Co., Ltd. | 80109218 |
| Camera | Canon | D70 |
| Dehydrator | DIAPATH | Donatello |
| Embedding machine | Wuhan Junjie Electronics Co., Ltd | JB-P5 |
| Pathology slicer | Leica | RM2016 |
| Frozen platform | Wuhan Junjie Electronics Co., Ltd | JB-L5 |
| Organizer | KEDEE | KD-P |
| Dyeing machine | DIAPATH | Giotto |
| oven | Labotery | GFL-230 |
| Glass slide | Servicebio | G6004 |
| Upright optical microscope | Nikon | NIKON ECLIPSE E100 |
| HE staining Imaging system | Nikon | NIKON DS-U3 |
| Ethanol | SCRC | 100092683 |
| Xylene | SCRC | 10023418 |
| HE dye solution set | Servicebio | G1003 |
| Neutral gum | SCRC | 10004160 |
| Ultra high performance liquid phase | Waters | Waters 2D UPLC |
| High resolution mass spectrometer | Thermo Fisher Scientific | Q Exactive HF |
| The chromatographic colum | Waters | ACQUITY UPLC CSH C18(1.7 μm,2.1*100 mm) |
| Low temperature high speed centrifuge | Eppendorf | Centrifuge 5430 |
| Vortex | Kylin-Bell Lab Instruments Co.,Ltd | QL-901 |
| Milli-Q apparatus | Millipore Corporation | Milli-Q Integral |
| Freezing vacuum concentrator | GENE COMPANY | Maxi Vacbeta |
| Tissue grinder | Shanghai Jingxin | JXFSTPRP |
| SPLASH Internal standards | Avanti  Polar Lipids | 330707, SPLASHTM Lipidomix Mass Spec Standard |
| Methanol | Thermo Fisher Scientific | A454-4 |
| Acetonitrile | Thermo Fisher Scientific | A996-4 |
| Dichloromethane | Thermo Fisher Scientific | D143-4 |
| Ammonium formate | Honeywell  Fluka | 17843-250G |
| Formic acid | DIMKA | 50144-50ml |

## 2 Supplementary Figures


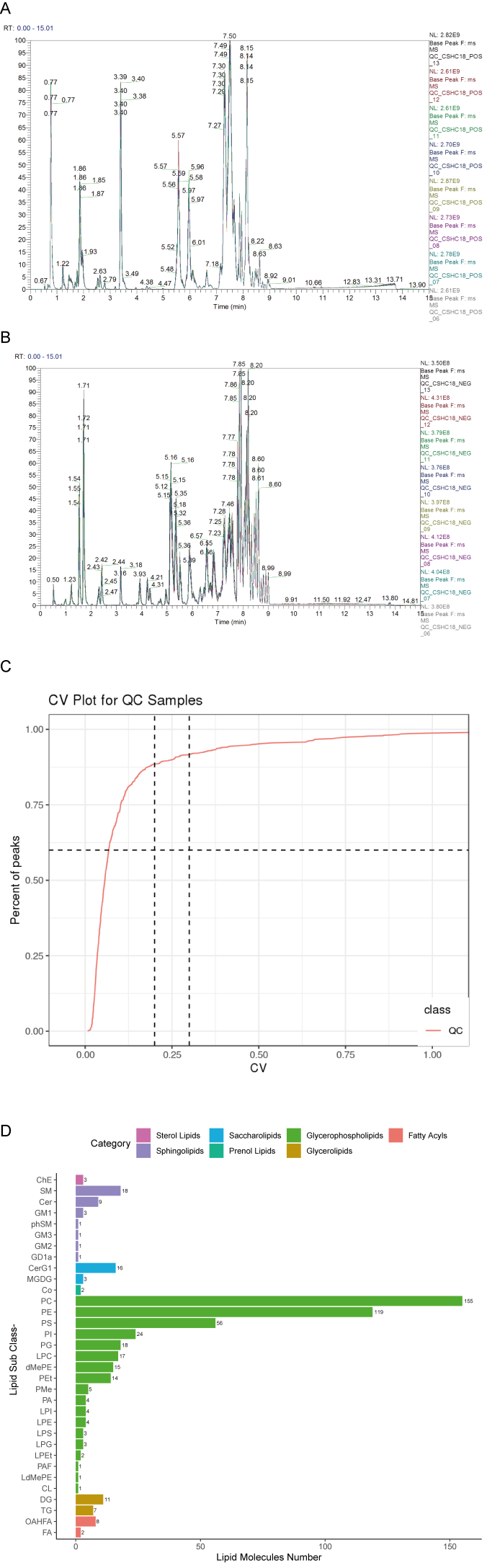


**Supplementary Figure Ⅰ Quality control and lipid identification of lipidomic data from hippocampus tissues of ApoE^-/-^ mice fed continuous high-fat diets combined with bound stimulation.** (A, B) BPC (base peak chromatogram) overlay maps of QC samples from hippocampus tissue (left: positive ion pattern; right: negative ion pattern). (C) CV distribution of lipid metabolites in QC samples from hippocampus tissue. (RSD_30_number(RSD(CV)<= 30%): the number of lipid metabolites in QC sample with RSD(CV) less than or equal to 30%; RSD_Ratio: the ratio of the number of lipid metabolites with RSD(CV) less than or equal to 30% to the number of all detected lipid metabolites in QC sample; Ratio>=0.6 means the data Ratio>=0.6 is satisfactory.). (D) Statistical plots of lipid subclasses and the number of corresponding lipid metabolites in hippocampus tissue. The vertical axis shows the lipid subclasses identified in this experiment, the horizontal axis shows the number of lipid metabolites identified in each lipid subclass, different colors indicate different lipid categories.


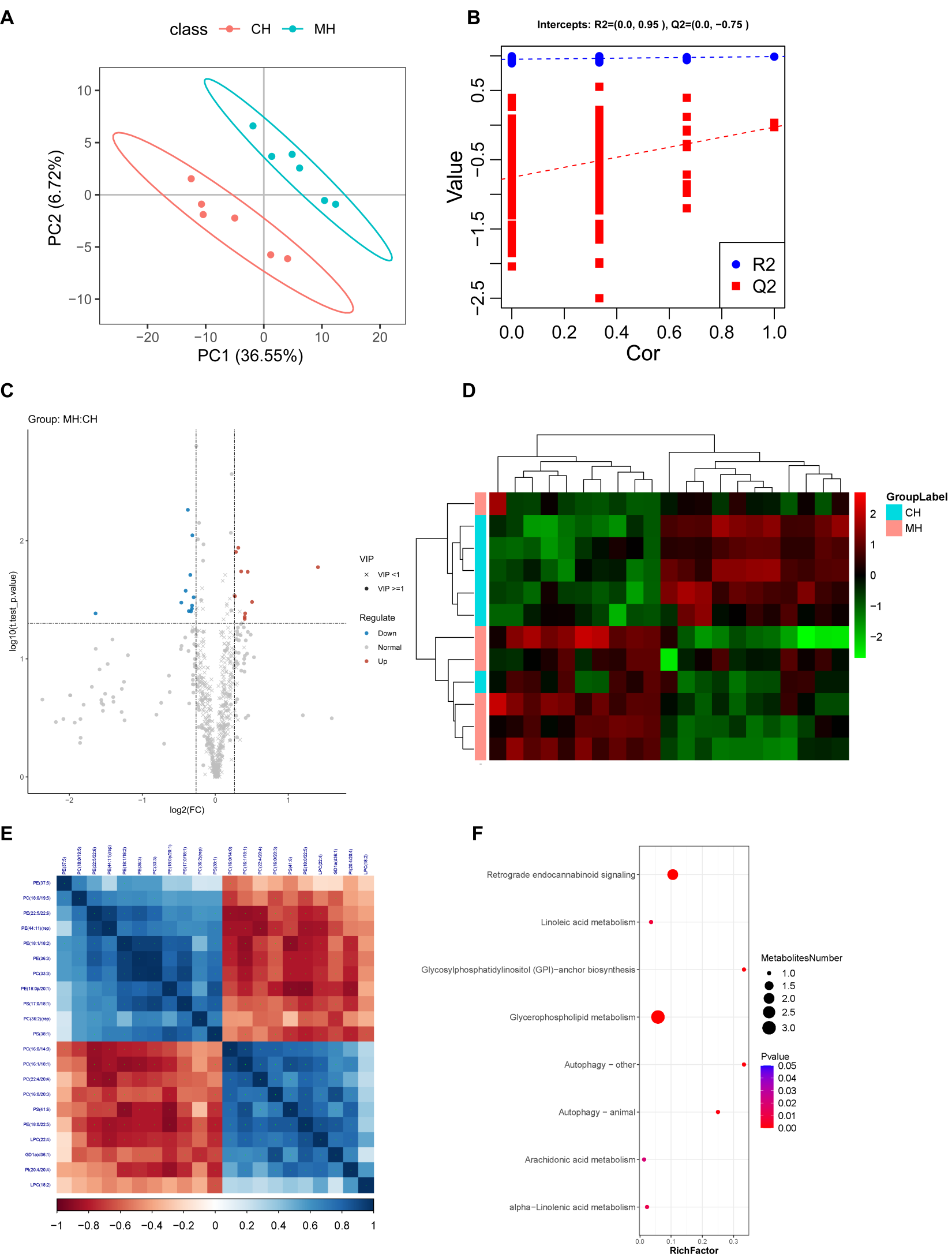


**Supplementary Figure Ⅱ Molecular analysis of differential lipids in the hippocampus tissues of ApoE^-/-^ mice fed a continuous high-fat diet combined with bound stimulation.** CH: Hippocampus results of NC group, MH: Hippocampus results of HFB group. (A) Score plots for the PLS-DA discriminant analysis model of differential lipids in hippocampus tissue. The horizontal axis is the first principal component and the vertical axis is the second principal component. Numbers in parentheses are the scores of that principal component, indicating the explanatory power of that principal component for the overall model. (B) Plots of response ranking tests for the PLS-DA analysis model of differential lipids in hippocampus tissue. The two rightmost points in the plot are the true R2Y and Q2 values of the model, respectively, and the remaining points are the R2Y and Q2 values obtained from a random permutation of the samples used. (C) Volcano maps of differential lipids in hippocampus tissue. Volcano maps were used to visualize the screened differential lipid metabolites. Blue is down-regulated significant differential lipid metabolites, red are up-regulated significant differential lipid metabolites, circles are lipid metabolites with VIP greater than or equal to 1, "×" are lipid metabolites with VIP less than 1, and non-significant lipid metabolites are grey. (D) The plot of differential lipid clustering analysis in hippocampus tissue. Each column in the graph represents a differential ion, each row represents a sample, and different colors indicate different intensities, with colors ranging from green to red, indicating low to high intensity. (E) Heat map of molecular correlations of differential lipids in hippocampus tissue, blue represents positive correlations, red represents negative correlations, the darker the color, the greater the linear correlation. "*" indicates a p-value<0.05 for a statistical test of the correlation coefficient. (F) Enrichment map of KEGG metabolic pathway of differential lipid metabolites in hippocampus tissue. The size of the dot represents the number of different lipid metabolites enriched in this pathway, the color represents the P-value, and the redder the color, the smaller the P-value.


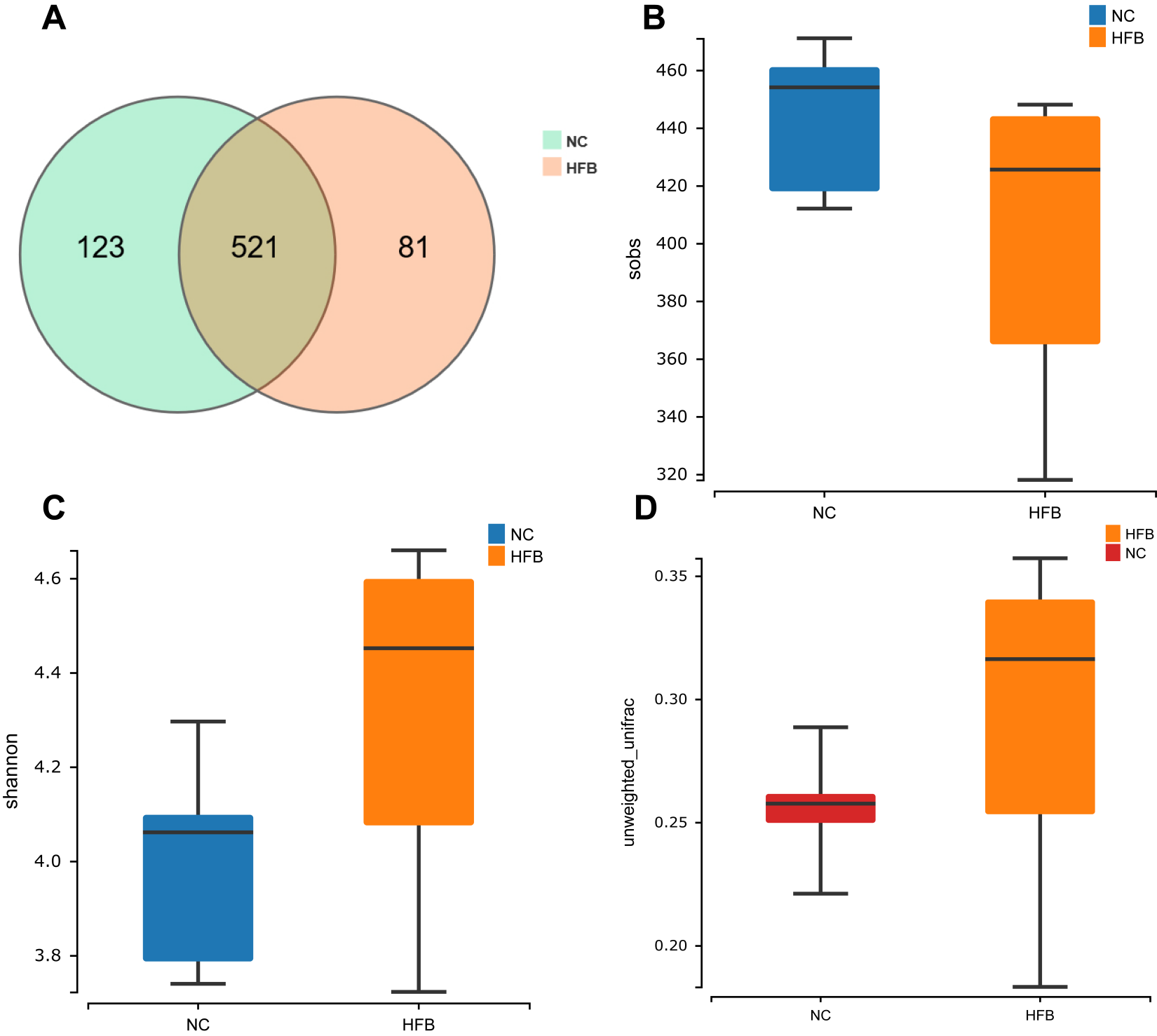


**Supplementary Figure Ⅲ Changes in intestinal flora in ApoE^-/-^ mice fed continuous high-fat combined with bound stimulation.** (A) Venn graph (B) Sobs index statistic graph (C) Shannon index statistic graph (D) Beta diversity inter-group variation graph.


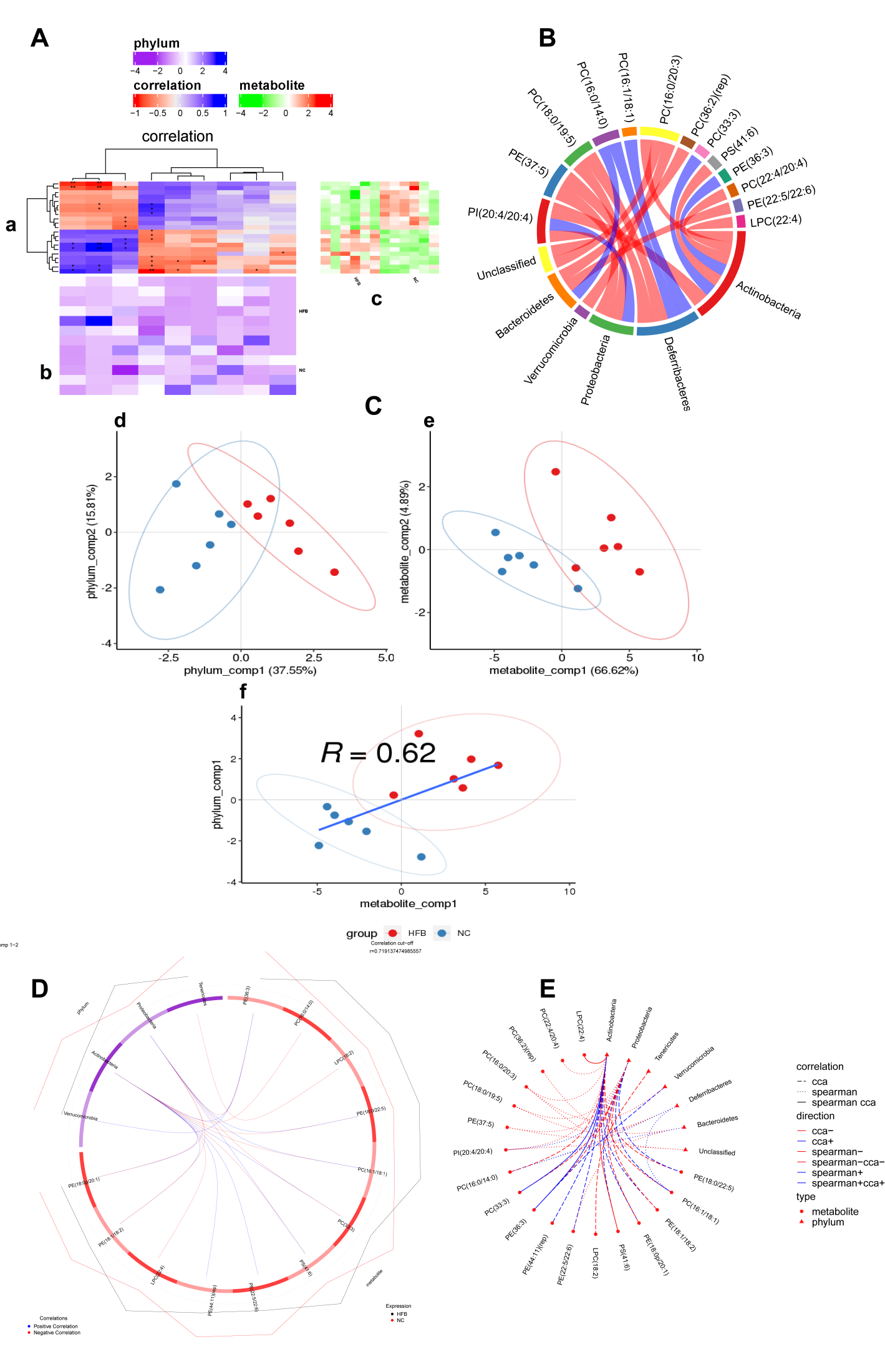


**Supplementary Figure Ⅳ Correlation analysis of differential metabolites and microbial taxa in the hippocampus tissue of ApoE^-/-^ mice fed continuous high-fat diets combined with bound stimulation.** (A) Heat map of the correlation between differential metabolites and microbial taxa in hippocampus tissue. (a) clustering heat map of correlation between differential metabolites and microbial taxa, horizontal coordinates are microbial taxa, vertical coordinates are differential metabolites, * indicates P value<0.05,** indicates P value<0.01; red indicates a negative correlation, blue indicates positive correlation, the darker the color, the stronger the correlation; (b) heat map of relative abundance of microbial taxa, rows represent samples, row names are group names, columns represent microbial taxa, those with column names are differential microbial taxa, from yellow to red indicate low to high relative abundance. (c) heat map of differential metabolite abundance, rows represent differential metabolites, columns represent samples, column names are group names; Pathway: metabolic pathways significantly enriched in differential metabolites, each color other than grey represents a metabolic pathway. The complex heat map is clustered based on the correlation coefficients in plot a. Microbial taxa with high correlation with differential metabolites are clustered together in plot b, while differential metabolites with high correlation with microbial taxa are clustered together in plot C. (B) Chord diagram of the correlation between differential metabolites and microbial taxa in hippocampus tissue. Each node represents a differential metabolite or microbial taxon, and the red arcs between nodes represent negative correlations and the blue arcs represent positive correlations. (C) Scatter plot of the correlations between differential metabolites of hippocampus tissue and microbial taxa. (d) scatter plot of microbial taxon components, the horizontal coordinate is the first component value, the vertical coordinate is the second component value; (e) scatter plot of differential metabolite components, the horizontal coordinate is the first component value, the vertical coordinate is the second component value; (f) scatter plot of Pearson correlation between differential metabolites and microbial taxon first components, the horizontal coordinate is the metabolic pathway first component value, the vertical coordinate is the microbial taxon first component value. A larger R indicates a higher degree of correlation between microbial taxa and the first component of the metabolic pathway. (d, e, f) Each point in the plot represents a sample, and the colours and ovals represent sample groupings; the greater the sample dispersion in different groups, the better the classification of that component value. (D) Ring plots of differential metabolite and microbial correlations in hippocampus tissue. The broken line on the periphery of the ring represents the abundance value of the differential metabolite and the microbial group in each group, the distance between the broken lines represents the difference between the groups, the upper right corner is the correlation coefficient threshold, and only the differential metabolites with the absolute value of the correlation coefficient greater than the threshold There is a connection with the microbial group. The blue connection represents a positive correlation, and the red connection represents a negative correlation. (E) Network diagram of a correlation between differential metabolites and microbial taxa in hippocampus tissue. Circles are metabolites, triangles are microbial taxa, red lines indicate negative correlations, blue lines indicate positive correlations, long dashed lines indicate typical correlations, short dashed lines indicate rank correlations and solid lines indicate typically and rank correlations.
